# Comparative analysis of *B-BOX* genes and their expression pattern analysis under various treatments in *Dendrobium officinale*

**DOI:** 10.1186/s12870-019-1851-6

**Published:** 2019-06-10

**Authors:** Yunpeng Cao, Dandan Meng, Yahui Han, Tianzhe Chen, Chunyan Jiao, Yu Chen, Qing Jin, Yongping Cai

**Affiliations:** 1 Key Laboratory of Cultivation and Protection for Non-Wood Forest Trees, Ministry of Education, Central South University of Forestry and Technology, Changsha, 410004 Hunan China; 2 School of Life Sciences, Anhui Agricultural University, Hefei, 230036 China

**Keywords:** *BBX*, *D. officinale*, Abiotic stress, qRT-PCR, Evolution

## Abstract

**Background:**

Studies have demonstrated that *BBX* (B-BOX) genes play crucial roles in regulatory networks controlling plant growth, developmental processes and stress response. Nevertheless, comprehensive study of *BBX* genes in orchids (Orchidaceae) is not well studied. The newly released genome sequences of *Dendrobium officinale* and *Phalaenopsis equestris* have allowed a systematic analysis of these important *BBX* genes in orchids.

**Results:**

Here we identified 19 (*DoBBX01*–*19*) and 16 (*PeBBX01–16*) *BBX* genes from *D. officinale* and *P. equestris*, respectively, and clustered into five clades (I-V) according to phylogenetic analysis. Thirteen orthologous, two *DoBBXs* paralogous and two *PeBBXs* paralogous gene pairs were validated. This gene family mainly underwent purifying selection, but five domains experienced positive selection during evolution. Noteworthy, the expression patterns of root, root_tips, stem, leaf, speal, column, lip, and flower_buds revealed that they might contribution to the formation of these tissues. According to the cis-regulatory elements analysis of *BBX* genes, qRT-PCR experiments were carried out using *D. officinale* PLBs (protocorm-like bodies) and displayed that these *BBX* genes were differentially regulated under AgNO_3_, MeJA (Methyl Jasmonate), ABA (abscisic acid) and SA (salicylic acid) treatments.

**Conclusions:**

Our analysis exposed that *DoBBX* genes play significant roles in plant growth and development, and response to different environmental stress conditions of *D. officinale*, which provide aid in the selection of appropriate candidate genes for further functional characterization of *BBX* genes in plants.

**Electronic supplementary material:**

The online version of this article (10.1186/s12870-019-1851-6) contains supplementary material, which is available to authorized users.

## Background

Zinc-finger proteins play important roles in regulate of plant growth development, and biotic and abiotic stress [1, 2]. Among them, the B-BOX zinc finger protein subfamily contains one or more B-BOX domains, which are composed of the conserved Cysteine (C) and Histidine (H), and stabilize its unique tertiary structure by binding with Zn-ions [3, 4]. The B-BOX domain might be involved in the interaction between zinc-finger proteins and other proteins [3, 5]. More than 1500 proteins, which containing B-BOX domain, were found in eukaryotes. Most of the B-BOX domains in animal cells were conjugated with the RING finger domain and coiled-coiled domain to form trivalent structural proteins or RBCC. In RBCC complex-mediated protein ubiquitination degradation pathway, B-BOX domain might be involved in substrate recognition process [6]. The B-BOX domain can be divided into two types, namely [C3(C/H)H2](BOX1(C-X2-X-X6–7-C-X2-X4–8-C-X2–3-C/H-X3–4-H-X5–10-H and [CHC(D/C)C2H2](B-box2(C-X2–4-H-X7–10-C-X1–4-D/C4–7-C-X2-C-X3–6-H-X2–5-H). Both of these two domains contain 7 or 8 conserved cysteines (C) and histidine (H) residues and together with two Zn-atoms to form a RING-like fold [7, 8].

In *Arabidopsis thaliana*, Khanna et al. (2009) identified 32 proteins containing B-BOX domain in N-terminal, named BBX1–32 [4]. As compared to animals, the B-BOX protein of *A. thaliana* had at least one B-BOX domains that interact with the Zn atom through the aspartic acid (Asp) residue [7, 9]. Although the B-BOX domain was considered to be involved in protein interactions, the function of B-BOX proteins in plants is not well clear. The B-BOX proteins STH3 (SALT TOLERANCE HOMOLOG03) and BBX22 of *A. thaliana* could interact with two key regulators of the light signaling pathway, HY5 and COPI, to regulate plant light-dependent developmental processes [7, 9–11]. In addition, STH3, BBX22 could be degraded by ubiquitination of COPI in vitro [7, 9]. The eight genes encoding double B-BOX domain proteins in *A. thaliana* were studied. It was proved that the expression of five *BBX* genes is regulated by the circadian rhythm [4]. Overexpression of *OsBBX25* gene in *A. thaliana* could enhance salt and drought tolerance of *A. thaliana* plants, and the expression of KIN1, RD29A and COR15 in transgenic plants is up-regulated under salt stress. *OsBBX25* might regulate the expression of stress response-related genes as a cofactor of transcriptional regulation, and then participate in plant response to abiotic stress [12, 13].

Orchids occupy about 10% of flowering plants, almost all over the world [14]. *D. officinale* and *P. equestris* belong to epiphytic orchids, of which *D. officinale* is also a valuable Chinese herbal medicine plant. The B-BOX gene family has been identified in several plants, such as *Pyrus bretschneideri*, *O. sativa*, and *A. thaliana* [4, 12, 15]. In addition, many studies have confirmed that B-BOX genes play key roles in plant growth and development, and responses to abiotic and biotic stresses. The draft of the *D. officinale* and *P. equestris* genome sequences were reported recently, respectively [16–18]. To further understand the B-BOX gene family in orchids, we respectively identified all 19 and 16 *BBX* members in *Dendrobium officinale* and *Phalaenopsis equestris*, and analyzed their phylogenetic relationships, gene structures, cis-regulatory elements, tissue expression patterns, as well as expression profiles under AgNO_3_, MeJA (Methyl Jasmonate), ABA (abscisic acid) and SA (salicylic acid) treatments. Our study will facilitate further functional studies of specific genes in the *BBX* family.

## Results

### The *BBX* gene family members in *D. officinale* and *P. equestris*

**Fig. 1:**
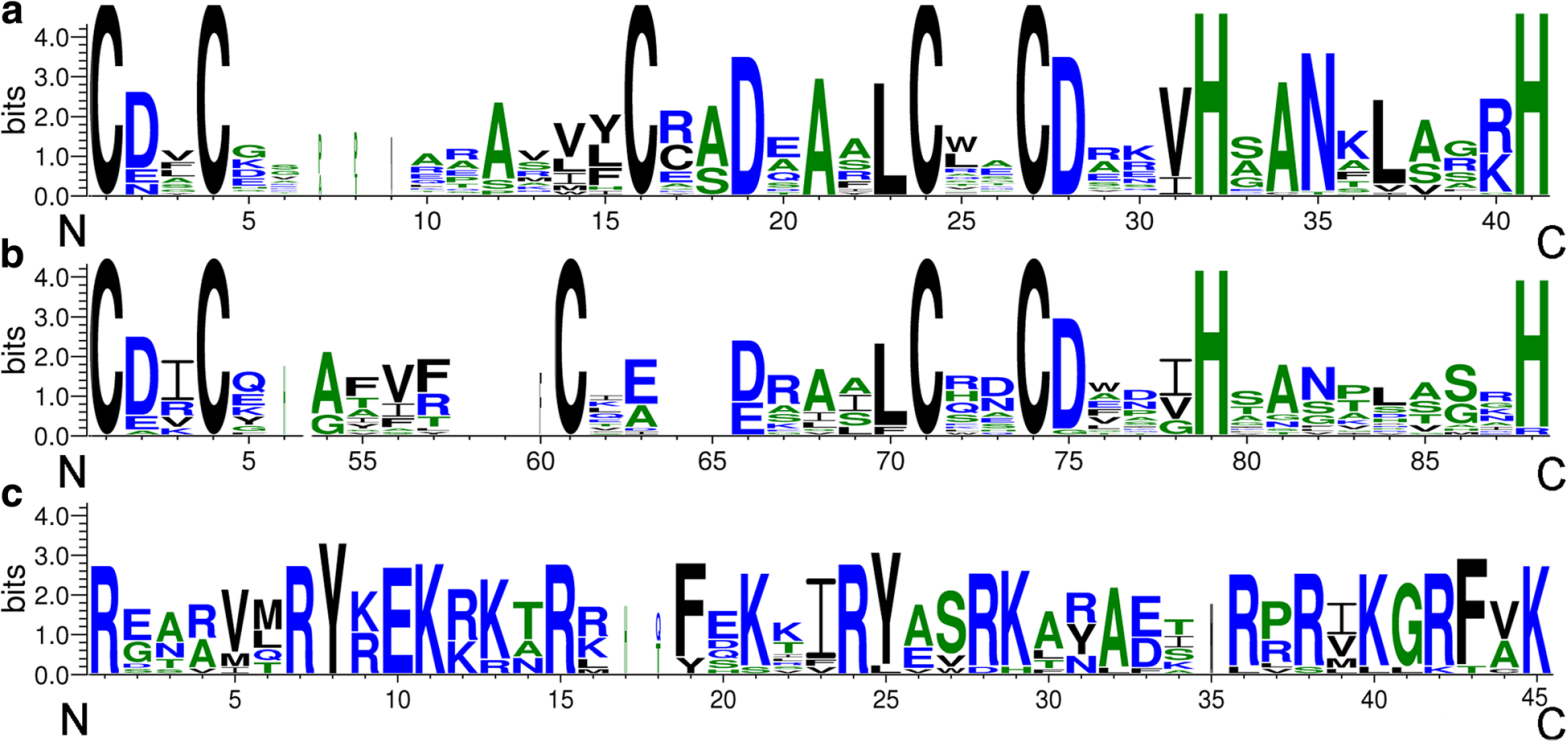
Domain composition of DoBBX and PeBBX proteins. a, b and c indicate the amino acid sequence alignment of the B-BOX 1, B-BOX 2 and CCT domain, respectively. The y-axis and x-axis indicated the conservation rate of each amino acid and the conserved sequences of the domain, respectively

A total of 24 and 22 B-BOX (BBX) coding protein sequences were identified in *D. officinale* and *P. equestris*, respectively, with the BLASTP program and HMMER software. However, some sequences not contained B-BOX domain (Fig. 1), five and six sequences from *D. officinale* and *P. equestris*, respectively, were excluded in this study. According to their scaffold positions, these genes were named *DoBBX01*-*DoBBX19* and *PeBBX01*-*PeBBX16*, respectively. The detailed information (gene name, gene identifiers, scaffold position, molecular weight, and theoretical isoelectric point) of each BBX was presented in Table 1. As shown in Table 1, the *BBX* genes showed large differences in terms of their theoretical isoelectric points and molecular weights. In *D. officinale*, the molecular weights ranging from 14,648.51 Da (DoBBX14) to 61,659.92 Da (DoBBX05) with an average molecular weight of 34,200.53 Da, with theoretical isoelectric points from 4.74 (DoBBX15) to 8.4 (DoBBX02). In *P. equestris*, the smallest and largest molecular weights were respectively 7492.76 Da (PeBBX14) and 49,942.19 Da (PeBBX06). Furthermore, the theoretical isoelectric points of these proteins ranged from 4.35 (PeBBX11) to 8.61 (PeBBX05), with an average of 6.24.

**Table 1:** The detailed information of *BBX* genes in *D. officinale* and *P. equestris*

Gene name	Gene ID	Location	3’	5’	PI	MW(Da)
*DoBBX01*	Dendrobium_GLEAN_10114110	scaffold1023	110,887	111,429	5.78	19,334.08
*DoBBX02*	Dendrobium_GLEAN_10103854	scaffold1579	145,245	147,705	8.4	16,408.94
*DoBBX03*	Dendrobium_GLEAN_10103350	scaffold1615	111,626	113,154	6.62	49,069.17
*DoBBX04*	Dendrobium_GLEAN_10087464	scaffold2703	47,629	52,112	8.05	23,160.00
*DoBBX05*	Dendrobium_GLEAN_10083337	scaffold3036	22,448	37,134	6.38	61,659.92
*DoBBX06*	Dendrobium_GLEAN_10082773	scaffold3055	36,993	49,949	5.97	47,501.90
*DoBBX07*	Dendrobium_GLEAN_10070006	scaffold4316	51,281	52,020	6.58	22,581.62
*DoBBX08*	Dendrobium_GLEAN_10066138	scaffold4764	5755	6951	5.17	31,455.23
*DoBBX10*	Dendrobium_GLEAN_10059161	scaffold5567	27,303	27,881	5.89	42,498.73
*DoBBX09*	Dendrobium_GLEAN_10059159	scaffold5567	17,687	22,526	4.8	40,018.87
*DoBBX11*	Dendrobium_GLEAN_10057235	scaffold5786	5947	12,621	5.74	21,072.85
*DoBBX12*	Dendrobium_GLEAN_10061306	scaffold6558	50,428	52,902	5.9	49,066.97
*DoBBX13*	Dendrobium_GLEAN_10048630	scaffold7094	5715	11,039	4.76	29,329.54
*DoBBX14*	Dendrobium_GLEAN_10048191	scaffold7130	45,763	46,503	6.49	14,648.51
*DoBBX15*	Dendrobium_GLEAN_10030321	scaffold10795	50	481	4.74	15,960.99
*DoBBX16*	Dendrobium_GLEAN_10027946	scaffold11444	5072	6516	5.61	32,830.90
*DoBBX17*	Dendrobium_GLEAN_10027874	scaffold11472	11,595	16,499	7.48	33,074.70
*DoBBX18*	Dendrobium_GLEAN_10027219	scaffold11757	21,929	22,994	6.06	30,682.53
*DoBBX19*	Dendrobium_GLEAN_10018021	scaffold15694	2698	3306	5.06	22,740.86
*PeBBX01*	PEQU_00678.1	Scaffold000002	12,666,119	12,668,574	6.02	35,945.50
*PeBBX02*	PEQU_00789.1	Scaffold000002	14,834,552	14,844,365	5.74	39,882.90
*PeBBX03*	PEQU_20117.1	Scaffold000008	219,001	219,795	5.13	29,606.44
*PeBBX04*	PEQU_30892.1	Scaffold000028	40,165	74,304	6.65	23,352.53
*PeBBX05*	PEQU_07418.1	Scaffold000202	1,196,612	1,197,568	8.61	35,776.22
*PeBBX06*	PEQU_26523.1	Scaffold000219	340,992	368,429	6.83	49,942.19
*PeBBX07*	PEQU_04684.1	Scaffold000224	4,007,418	4,008,506	6.02	37,255.94
*PeBBX08*	PEQU_07681.1	Scaffold000297	942,007	946,699	5.5	37,633.50
*PeBBX09*	PEQU_15996.1	Scaffold000411	691,452	710,139	6.85	47,215.68
*PeBBX10*	PEQU_14525.1	Scaffold000413	746,717	747,431	7.93	21,747.15
*PeBBX11*	PEQU_32256.1	Scaffold000584	11,714	12,343	4.35	23,138.88
*PeBBX12*	PEQU_17923.1	Scaffold001081	902,228	902,798	6.57	18,004.64
*PeBBX13*	PEQU_41941.1	Scaffold001148	10,708	12,869	5.65	26,279.96
*PeBBX14*	PEQU_41825.1	Scaffold197624	33	242	6.68	7492.76
*PeBBX15*	PEQU_31514.1	Scaffold210877	95,748	102,447	5.76	20,454.13
*PeBBX16*	PEQU_36421.1	Scaffold233868	116,085	131,657	5.53	45,564.70

### Protein domain(s) and phylogenetic analysis

**Fig. 2:**
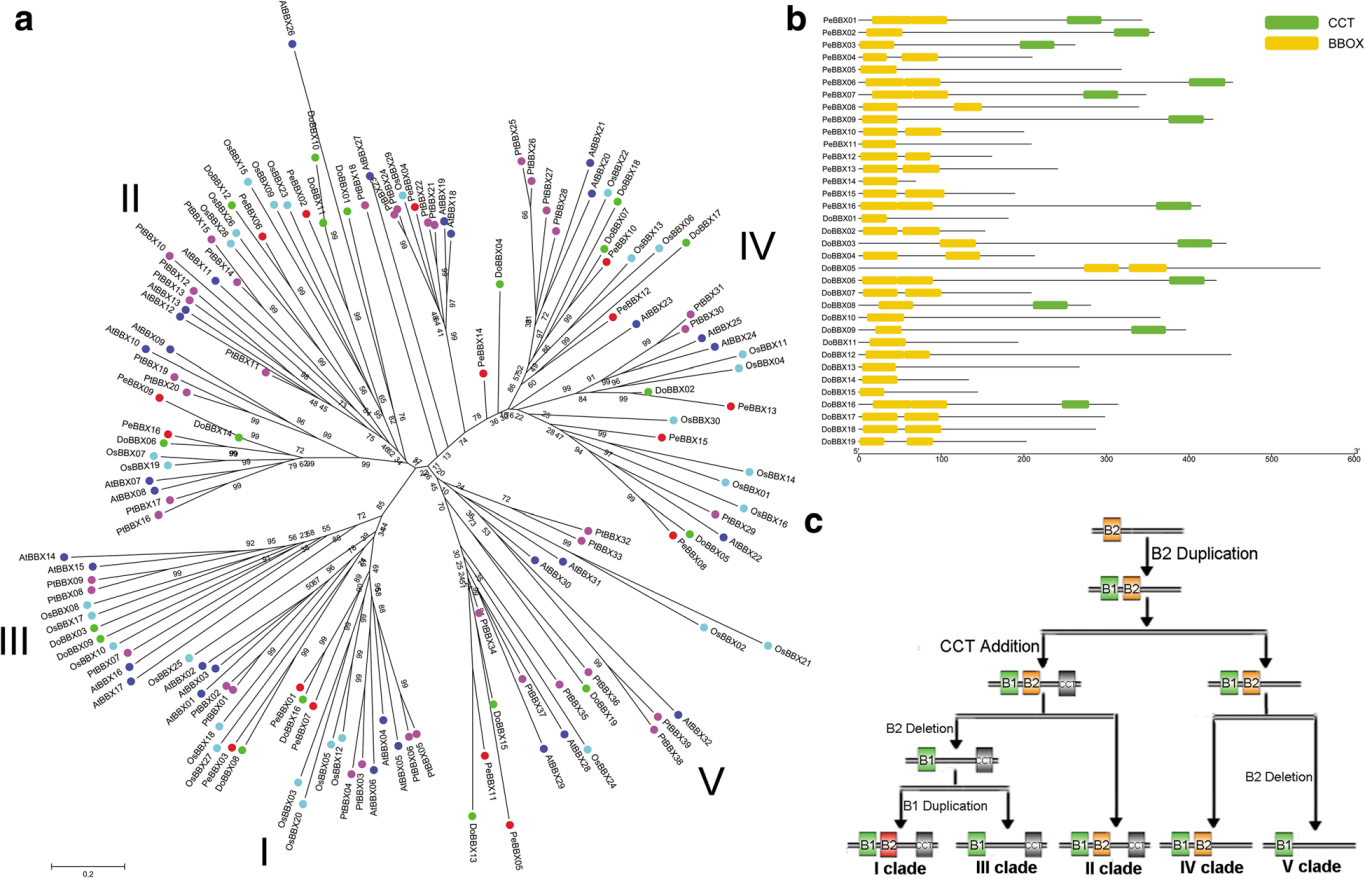
Phylogenetic relationships and domain structures of the *BBX* genes. **a**. Phylogenetic analysis of *BBX* genes in *Populus trichocarpa, A. thaliana*, *O. sativa*, *D. officinale* and *P. equestris*, (**b**). Domain structures of the BBX proteins in *D. officinale* and *P. equestris.* CCT and BBX indicated CCT and B-BOX domains, respectively. **c**. A hypothetic model for the *BBX* evolutionary trajectory in *D. officinale* and *P. equestris*. B1, B2, CCT indicated B-Box 1, B-Box 2, and CCT domains, respectively

The domains logos of the CCT domain and B-BOX domains (including B-BOX1 and B-BOX2 domain) of these *BBX* proteins were generated and were shown in Fig. 1 and Fig. 2. Among the 35 BBXs, we scanned that six genes had two conserved B-BOX domains and a CCT domain. Six and six proteins contained only one B-BOX domain, or one B-BOX plus a CCT domain, respectively, while the most numbers of BBX having two B-BOX domains. Among the three domains, we found that some conserved amino acid residues were similar, but not exactly the same. In the B-BOX domains, there are 5 conserved Cys residues in the Cys-X-X-Cys motifs, of which 4 are absolutely conserved. Additionally, there are several other conserved amino acid residues, such as two His, Asp, Ala, and Asn, as shown in Fig. 1. In the CCT domain, the consensus sequence is R-XXXXX-R-Y-X-E-K-XXX-R-XXX-K-XX-R-Y-XX-R-K-XX-A-XX-R-X-R-X-K-G-R-F-X-K.

**Fig. 3:**
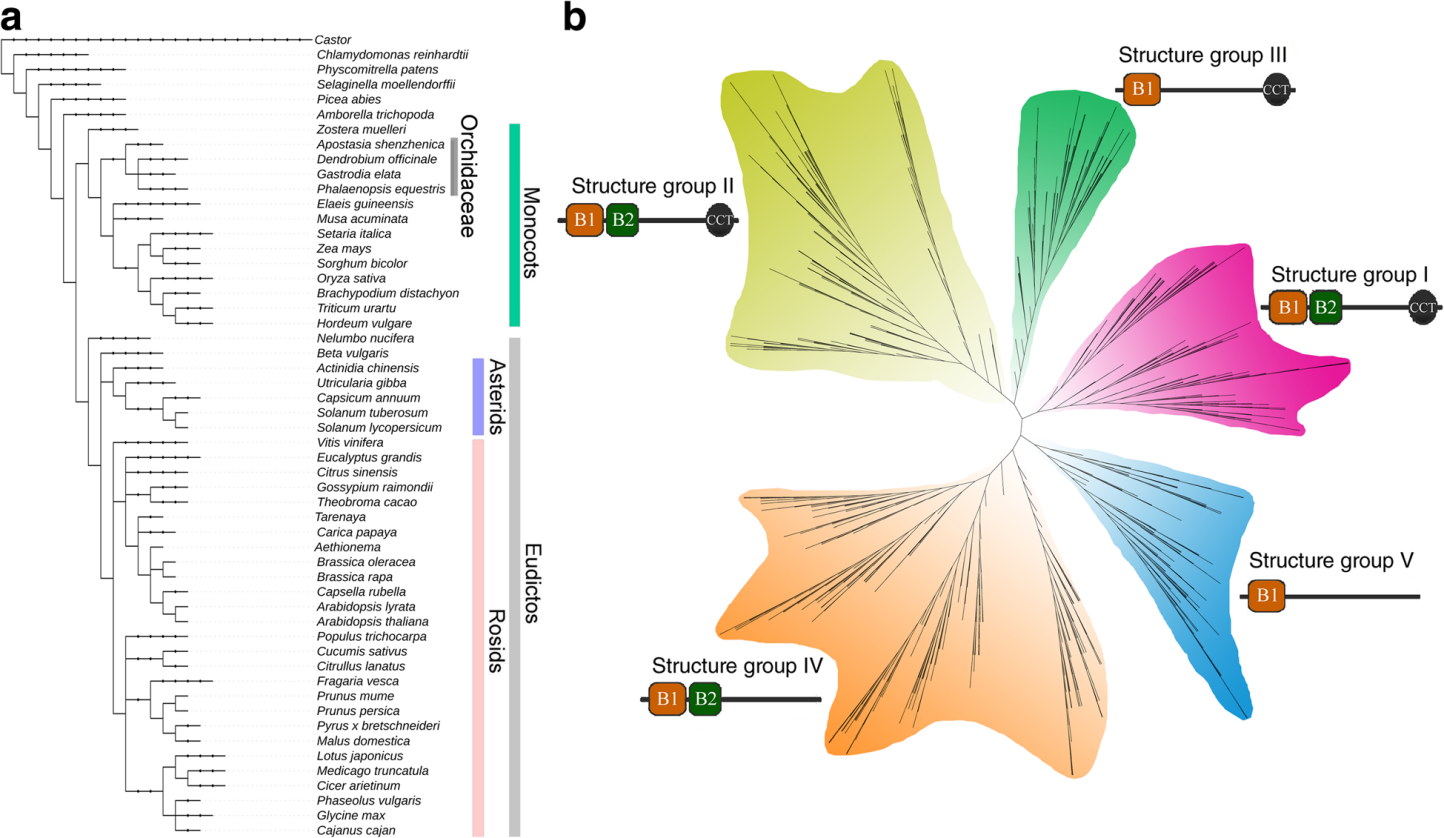
Species used in this study (**a**) and Maximum-likelihood gene tree (**b**) for the *BBX* gene family. The HMM (hidden Markov model) profile of BBX domain (Pfam00643) was used to identify for all of BBXs in these plant genomes with HMMER 3.0 software. Each putative BBX protein was further examined the presence of B-BOX domain by submitting them to SMART database, Pfam and InterProScan, respectively. The Maximum likelihood (ML) tree was generated by FastTree software with JTT model. A total of 1340 *BBX* genes were divided into four clades and were distinguished by different colors and legends

To analyze the evolutionary relationships and divergences of the *BBX* genes, the phylogenic tree, including *BBX* genes from *Populus trichocarpa, A. thaliana*, *O. sativa*, *D. officinale* and *P. equestris*, were generated (Fig. 2a). All sequences could be clustered into five clades and named as clade I–V in the phylogenetic tree following the previous articles [4, 15]. The *BBX* genes in clade I, clade II and clade III contained additional CCT domain, with one B-BOX domain in clade III, as well as two B-BOX domains in clades I and II. The remaining clade IV and clade V contained two and one B-BOX domain(s), respectively, but not have CCT domain. To further determine the evolutionary relationship of the B-BOX domain in the plant genomes (Fig. 3), 54 plant genomes covering angiosperms, gymnosperms, mosses and green algae were analyzed to generate maximum likelihood (ML) tree by using FastTree software [19]. Based on the phylogenetic analysis (Fig. 3b), a hypothesis evolutionary relationship of the B-BOX domain was proposed in this study (Fig. 2c). The early BBX sequences of the plant genomes originally contained only one B-BOX domain, and then the B-BOX domain had a duplication event during the evolution process, which was consistent with the fact that most of the green algae (i.e. *Chlamydomonas reinhardtii*) only had a single B-BOX domain. Remarkably, the CrBBX1 from an alga contained double B-BOX domain indicated that the first B-BOX duplication event occurred before green plants colonized the land [20]. A deletion event of the B-BOX domain in early BBX sequences belonging to the clade IV rises to a BBX sequence with a single B-BOX domain, which is a characteristic of the clade V. Later, the CCT domain was added at the C-terminus to generate a BBX protein with a double B-BOX and a CCT domain, which were early BBX member and belong to clade II. Among the clade II, some BBX members had a deletion event of the B-Box 2 domain resulted in the BBX proteins containing only one B-BOX and CCT domain (i.e. clade III). A duplication event of the B-Box 1 domain of an early BBX protein belonging to the clade II could have been a BBX precursor of clade I, generating BBX sequences with two B-BOX domains and one CCT domain. This proposed hypothesis was supported by similarities and differences in these domain sequences (Fig. 2c and Fig. 3b). For example, the B-Box 2 domain in clade I had a great difference compared with the sequence of the clade II and IV. These changes in the CCT and B-BOX domains resulted in the origin of different clades, which appeared in the early stages of plant genome evolution with retained the biological functions of the B-BOX domain.

### Evolutionary patterns and gene structure analysis

**Fig. 4:**
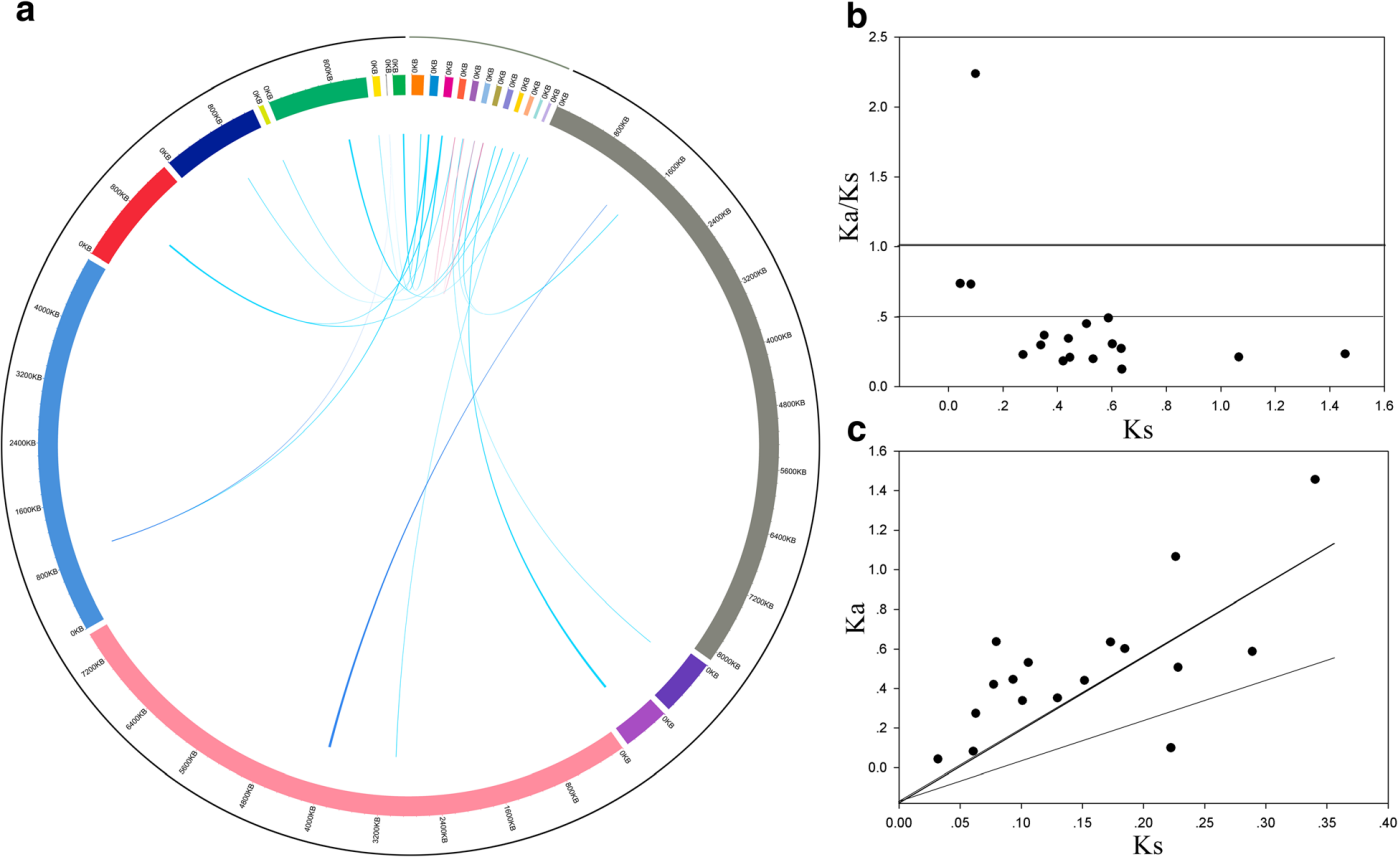
The analysis of homologous gene pairs in the *BBX* gene family. **a** Identification of paralogs and orthologs in *D. officinale* and *P. equestris.* Green, bule and red lines indicated orthologs gene pairs (Do-Pe), paralogs gene pairs (Pe-Pe), and paralogs gene pairs (Do-Do), respectively. **b** and **c** The distribution of Ka/Ks, Ks, Ka values of homologous gene pairs. The slopes of Ka/Ks =1 and 0.5 were represented by black and gray lines, respectively

To further understand the evolutionary patterns of *BBX* genes, we performed the analysis of orthologous and paralogous relationships among *D. officinale* and *P. equestris* genome. In the present study, two paralogs (Do-Do) in *D. officinale*, two paralogs (Pe-Pe) in *P. equestris*, and thirteen orthologs (Do-Pe) between *D. officinale* and *P. equestris* were identified by using the OrthoMCL software (Fig. 4a). Previous studies have shown that orchids share a genome-wide duplication event (the value of Ks is approximately equal to one) [14]. Subsequently, the Ka, Ks, and Ka/Ks of all homologous gene pairs were calculated (Additional file 1: Table S1). The Ks value of *PeBBX01*-*PeBBX07* and *DoBBX07*-*DoBBX18* was 1.0658 and 1.4562, respectively, indicating these gene pairs were derived from genome-wide duplication events by shared *D. officinale* and *P. equestris*. The Ks values for the other paralogs, including *PeBBX08*-*PeBBX14* and *DoBBX10*-*DoBBX11*, were 0.0433 and 0.0827, respectively, suggesting that they are derived from the ancient duplication events. According to the Ka/Ks value distributions (Fig. 4b and c) the homologous could divide into three classes. Nine homologous gene pairs had Ka/Ks values below 0.3, seven homologous gene pairs had Ka/Ks values between 0.3–1, and the remaining one gene pair (*DoBBX05*-*PeBBX08*) had ratios greater than 1. These data indicated that the most of *BBX* homologous gene pairs had undergone strong purifying selection during evolution. To further insight into the Ka/Ks value of each gene pair, we performed sliding-window analysis among all homologous pairs (Additional file 1: Figure S1). Based on this analysis, the majority of coding regions had Ka/Ks values were far below 1, but one or several distinct peaks (i.e. Ka/Ks value is greater than 1). The domains of the majority of BBXs commonly contained lower Ka/Ks values than the regions outside of them (i.e. peaks), which consistent with functional constraints being dominant in these domains. Combining with the above analysis, this *BBX* gene family mainly underwent purifying selection during evolution in *D. officinale* and *P. equestris* genome.

The evolution of multigene families could be driven by structural diversity [21]. In the present study, we constructed the exon-intron organization maps to survey the structural diversity of *BBX* genes (Additional file 1: Figure S2 and Figure S3). The 35 members of *BBX* gene family contained a variable number of exons, ranging from 1 to 7. Furthermore, we found that two *BBX* genes contained seven exons, two genes contained five exons and six genes contained four exons, seven genes contained three exons and ten genes contained two exons, while remaining eight genes only contained one exon. This phenomenon suggested that the *BBX* gene family has undergone both exon gain and loss during evolution, which might be able to further explain the functional differences of closely related *BBX* homologous genes. Subsequent the exon-intron structure of the *BBX* homologous gene pairs were further analyzed. Out of 17 gene pairs, the number of exons in the fifteen gene pairs had changed (Additional file 1: Figure S3), such as *DoBBX02*-*PeBBX13*, *DoBBX10*-*DoBBX11* and *PeBBX08*-*PeBBX14*. These divergences might be the result of single intron loss or gain events during evolution.

### Cis-acting element analysis

**Fig. 5:**
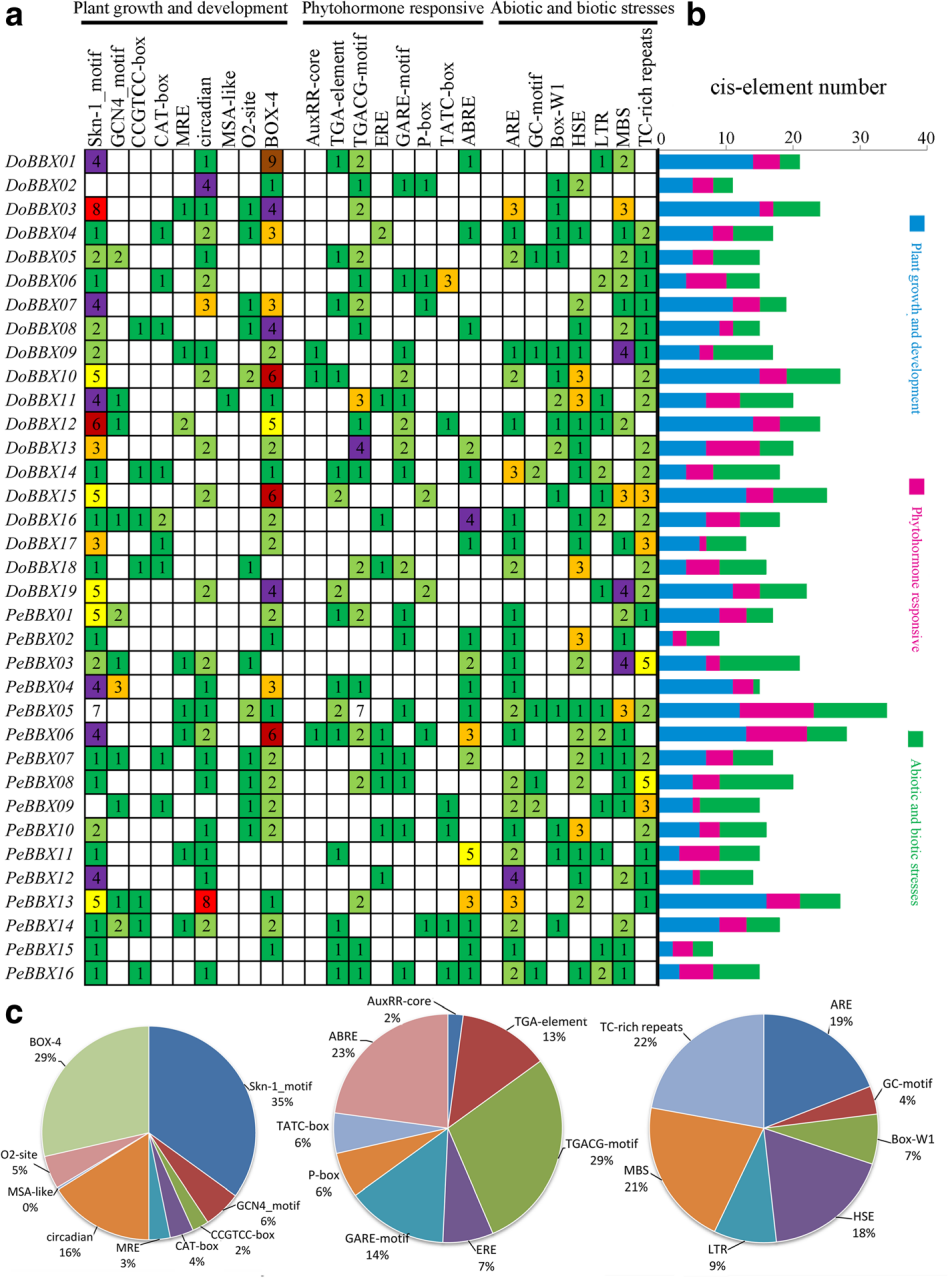
Investigation of cis-acting element numbers in all *BBX* genes of *D. officinale* and *P. equestris*. **a** The different colors and numbers of the grid indicated the numbers of different promoter elements in these *BBX* genes. **b** The different colored histogram represented the sum of the cis-acting elements in each category. **c** Pie charts of different sizes indicated the ratio of each promoter element in each category, respectively

Cis-regulatory elements play critical roles in regulatory networks controlling plant growth and development, including multi stimulus-responsive genes, and determining the tissue-specific or stress-responsive expression profiles of genes were closely associated with cis-elements in their promoter regions. Using the PlantCARE database, we identified three category cis-elements, including plant growth and development, biotic and abiotic stress responses and phytohormone responses in the promoter regions (Fig. 5). In growth and development category, cis-acting elements were found extensively in the promoter regions, including Skn-1-motif and GCN4_motif required for endosperm expression, CAT-box and CCGTCC-box for meristem expression, O2-site for zein metabolism regulation, MRE and Box 4for light responsiveness, and other cis-acting elements. Among these cis-acting elements, 98 Skn-1-motifs were identified and these motifs comprised the largest portion of the first category (Fig. 5). In the phytohormone responsive category, the TGA-element and AuxRR-core for auxin responsive, GARE-motif, P-box and TATC-box for gibberellin-responsive elements, and ERE for ethylene responsive were identified in eighteen *BBX* genes at most. Notably, the most common motif was the TGACG-motif cis-acting elements associated with MeJA-responsiveness, accounting for 29% of the scanned hormone responsive motifs (Fig. 5). Followed by ABRE cis-acting element, which was related to ABA; it accounted for 23% and appeared 32 times. In the last category, various stresses-related elements, such as ARE (anaerobic induction), Box-W1 (fungal elicitors), HSE (heat stress), TC-rich repeats (stress responses) and GC-motif (anoxia), were observed. Our data suggested that *BBX* genes might respond to abiotic stresses and had the potential to improve abiotic stress responses.

### The organ-specific expression profiling of *D. officinale BBX* genes

**Fig. 6:**
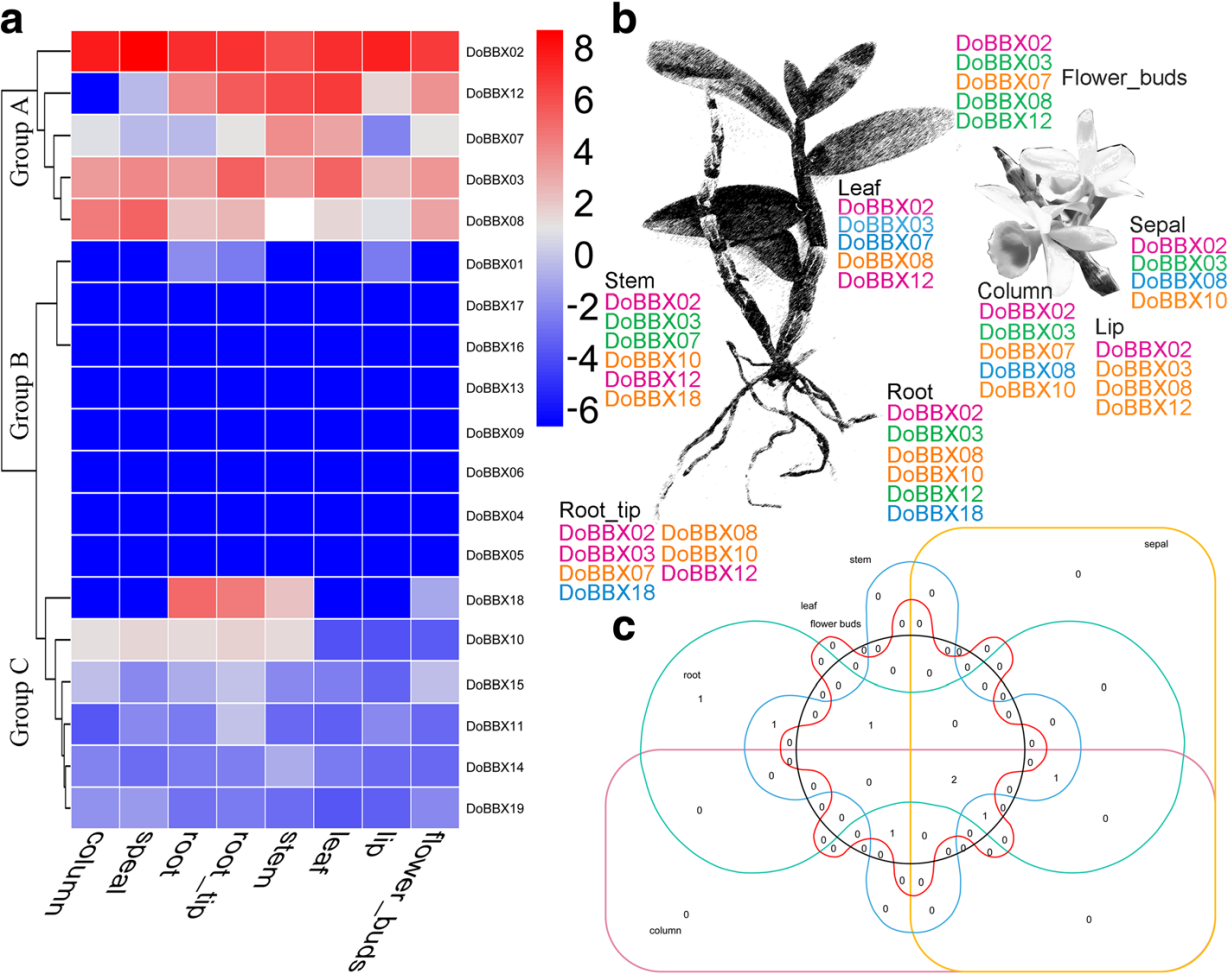
Expression pattern of *DoBBX* genes. **a** Organ-specific expression pattern of *DoBBX* genes in eight tissues: root, root_tips, stem, leaf, speal, column, lip and flower_buds. Blue and red indicated lower and higher transcript abundance, respectively. **b** Identification of highly expressed *BBX* genes in *D. officinale*. According to the previous studies [22], yellow, green, blue, red indicated low (1–6.8 FPKM), mid-low (6.8–17.5 FPKM), mid-high (17.5–44.7 FPKM), and high (44.7–17,092 FPKM) expression, respectively. **c** Venn diagram of *DoBBX* genes in different tissues

To further understand the dynamic gene expression of *BBX* gene family members in *D. officinale*, we performed the overall analysis of gene expression profiles in eight tissues (root, root_tips, stem, leaf, speal, column, lip and flower_buds). Based on the expression pattern in eight tissues, these *DoBBX* genes exhibited distinct organ-specific expression, and further divided into three groups (Fig. 6a). In the group A, five genes (*DoBBX02*, *− 03*, *− 07*, *− 08* and *− 12*) presented modest overall expression in all eight organs, suggesting that these *DoBBX* genes may play important roles in the formation of these tissues. Out of 19 genes, eight *BBXs* were classified into group B due to they were basically not expressed in these eight tissues. In group C, the remaining six genes shared similar lower expression in these tissues. Remarkably, not all homologous gene pairs exhibit similar patterns of expression, such as *DoBBX07* had highest transcript abundance in the stem and/or leaf, but the expression of its paralog, *DoBBX18*, was lowest in leaf and highest in root and/or root_tips. Additionally, several genes that were highly expressed in the flower organs have also been identified, including *DoBBX02*, *DoBBX07*, *DoBBX08* and *DoBBX12* (Fig. 6b and c)*.* These highest expression profiles of *DoBBXs* suggested that these genes might be indirectly or directly involved in the development and/or formation of reproductive organs. These results were also confirmed in *AtBBXs* (i.e. Arabidopsis *BBX* genes).

### Regulation of the expression of *D. officinale BBX* genes by abiotic stresses

A variety of abiotic and biotic stresses could affect a plant’s health and growth, and finally influence the regulation of a series of stress-related genes [23]. Therefore, it is very important to clarify the regulatory pathways and master regulators of stress responses in *D. officinale*. To better understand the stress responses involving the *D. officinale BBX* genes, the qRT-PCR experiments were used to analysis their expression under AgNO3, MeJA (Methyl Jasmonate), ABA (abscisic acid) and SA (salicylic acid) treatments.

**Fig. 7:**
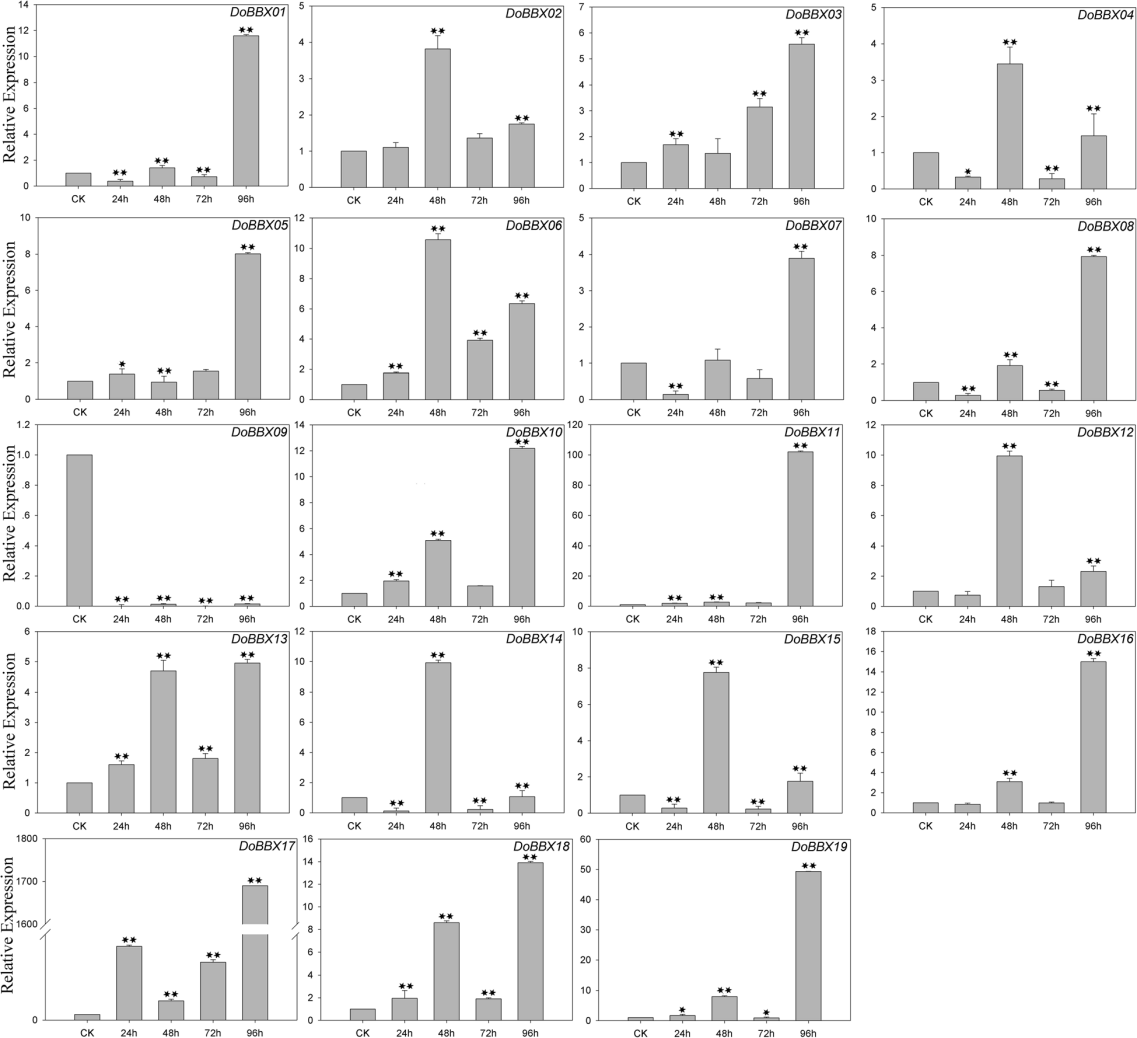
Expression patterns of *DoBBX* genes in *D. officinale* under ABA treatment stresses as determined by qRT-PCR experiment. The x-axis indicated the time course of each stress treatment, and the y-axis represented the relative expression level. Mean values and standard deviations (SDs) indicated by error bars. ** significant difference (*P* < 0.01), * significant difference at *P* < 0.05

**Fig. 8:**
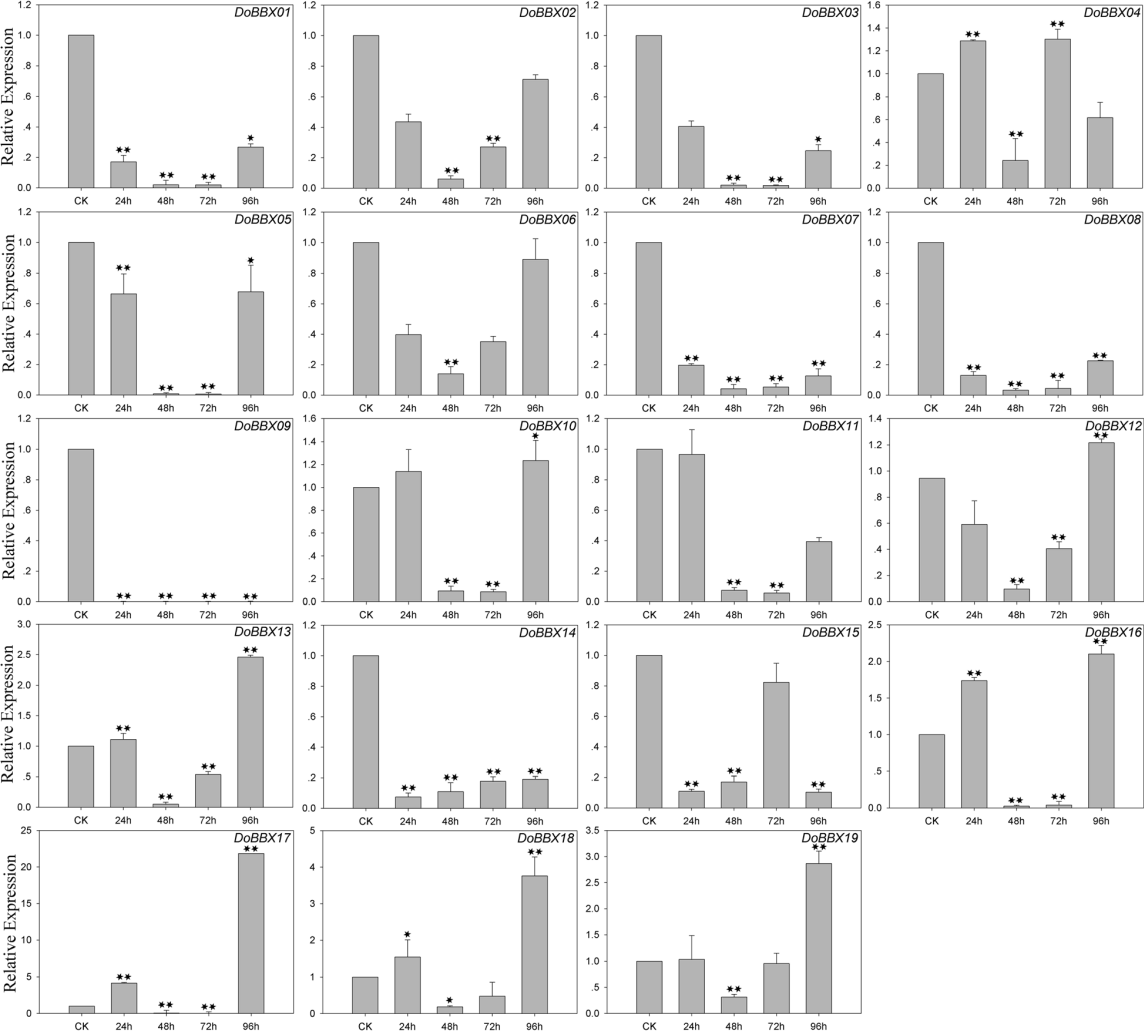
Expression patterns of *DoBBX* genes in *D. officinale* under SA treatment stresses as determined by qRT-PCR experiment. The x-axis indicated the time course of each stress treatment, and the y-axis represented the relative expression level. Mean values and standard deviations (SDs) indicated by error bars. ** significant difference (*P* < 0.01), * significant difference at *P* < 0.05

**Fig. 9:**
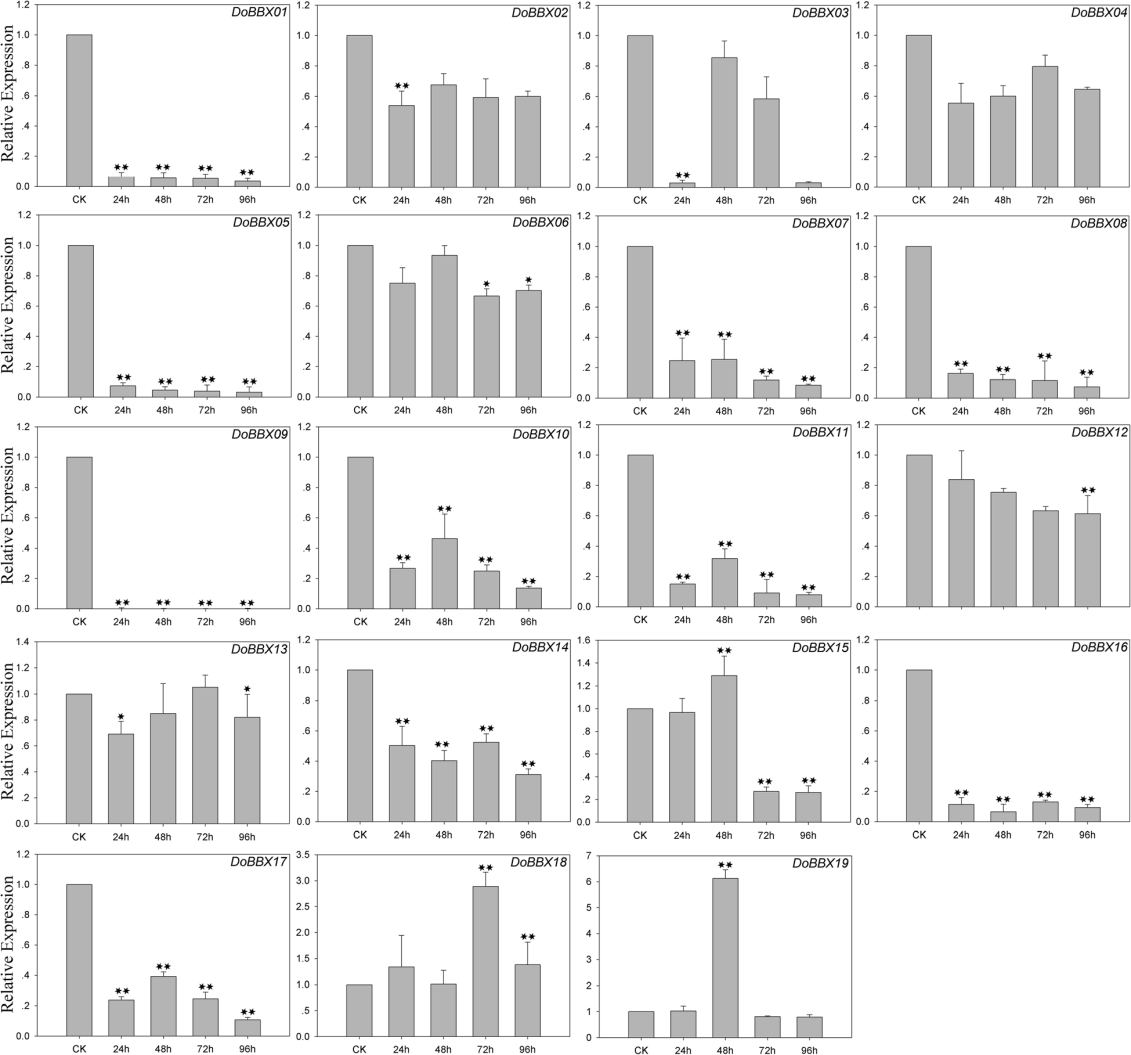
Expression patterns of *DoBBX* genes in *D. officinale* under AgNO_3_ treatment stresses as determined by qRT-PCR experiment. The x-axis indicated the time course of each stress treatment, and the y-axis represented the relative expression level. Mean values and standard deviations (SDs) indicated by error bars. ** significant difference (*P* < 0.01), * significant difference at *P* < 0.05

**Fig. 10:**
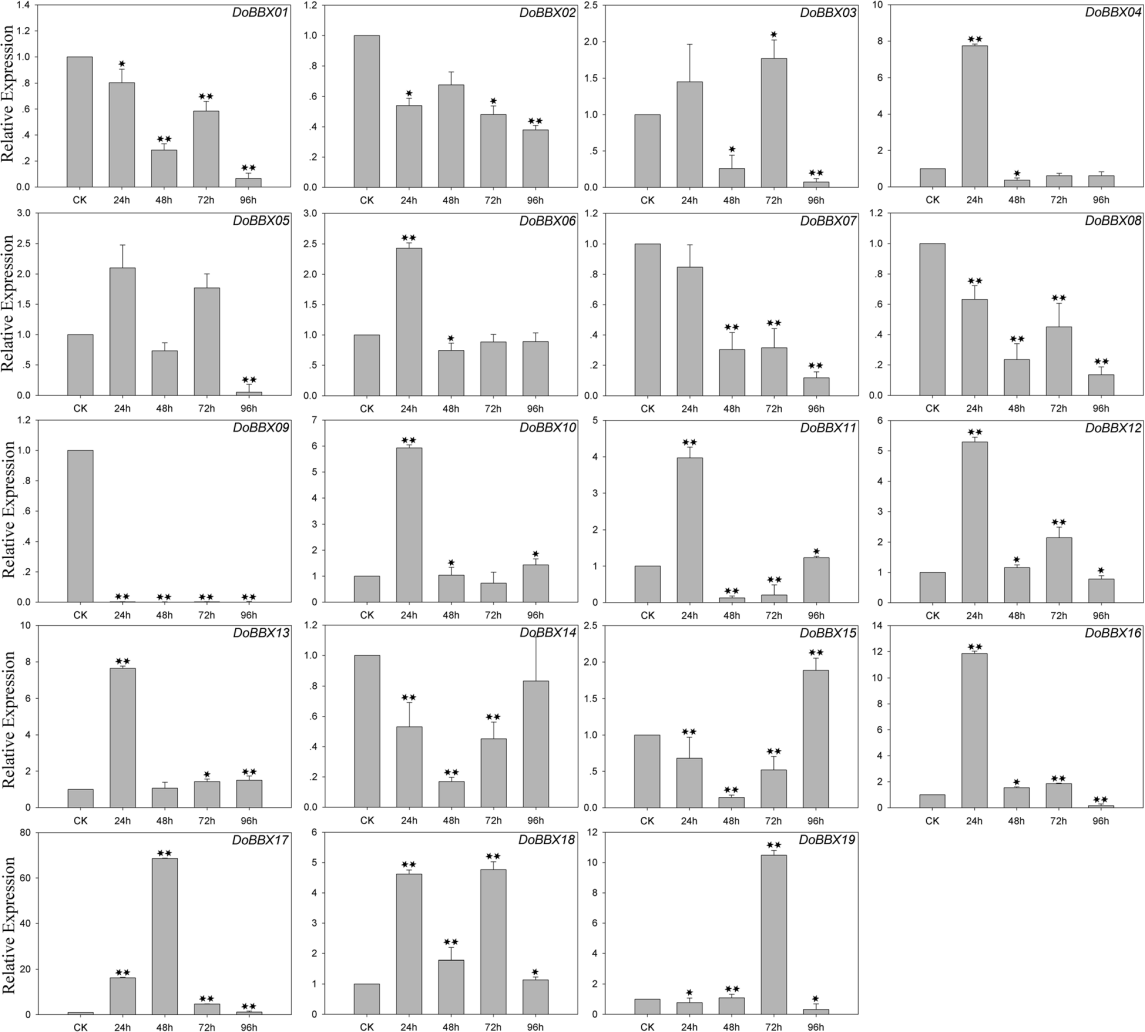
Expression patterns of *DoBBX* genes in *D. officinale* under MeJA treatment stresses as determined by qRT-PCR experiment. The x-axis indicated the time course of each stress treatment, and the y-axis represented the relative expression level. Mean values and standard deviations (SDs) indicated by error bars. ** significant difference (*P* < 0.01), * significant difference at *P* < 0.05

In the ABA treatment, eight *DoBBX* genes were up-regulated to different degrees by ABA treatment (Fig. 7). The exception was *DoBBX09*, which was obviously significantly and rapidly down-regulated at all time points. Among these *DoBBXs*, we found that the highest expression levels of *DoBBX01*, *DoBBX03*, *DoBBX05*, *DoBBX07*, *DoBBX08*, *DoBBX10*, *DoBBX11*, *DoBBX13*, *DoBBX16*, *DoBBX17*, *DoBBX18* and *DoBBX19* occurred 96 h after treatment: *DoBBX17* and *DoBBX11* were strongly up-regulated (by more than 1700-fold and 100-fold, respectively). The expressions of seven *DoBBX* genes (*DoBBX02*, *DoBBX04*, *DoBBX06*, *DoBBX12*, *DoBBX14* and *DoBBX15*) peaked at 48 h; *DoBBX06* and *DoBBX14* showed the greatest up-regulation by more than10-fold. Additionally, we found that the paralogous gene pairs contained similar expression patterns. For example, both *DoBBX07*-*DoBBX18* and *DoBBX10*-*DoBBX11* presented the same trend after at 48 h, with their highest levels at 97 h under ABA treatment. In the SA treatment, eleven *DoBBX* genes presented increased expression levels to different degrees (Fig. 8). Four of nineteen *DoBBX* genes were significantly up-regulated at the last time point (96 h), such as *DoBBX17* were up-regulated by more than 20-fold. Under AgNO_3_ treatment, fifteen *DoBBX* genes were obviously rapidly and significantly down-regulated at all time points. The remaining four *DoBBX* genes had highest expression levels at 48 h under AgNO_3_ treatment (Fig. 9). At MeJA treatment, the expression levels of *DoBBX04*, *DoBBX06*, *DoBBX10*, *DoBBX11*, *DoBBX12*, *DoBBX13*, *DoBBX15* and *DoBBX17* were strongly up-regulated at 24 h, such as *DoBBX16* was up-regulated by more than 10-fold. The *DoBBX03* and *DoBBX19* were up-regulated at 72 h (by more than 1.5-fold and 10-fold, respectively), while the *DoBBX17* was significantly up-regulated at 48 h (by more than 65-fold) under MeJA treatment (Fig. 10). The remaining *DoBBX* genes were down-regulated throughout the entire experimental period, including *DoBBX01*, *DoBBX02*, *DoBBX07*, *DoBBX08*, *DoBBX09*, and *DoBBX14*.

## Discussion

Most transcription factors are specific to plants and play an important role for plant growth and development [4, 12, 15]. As a class of transcription factors, members of the *BBX* family encode proteins have B-BOX domain(s), and some of them contain additional CTT domain. In this study, we characterized and identified 19 and 16 B-BOX genes in *D. officinale* and *P. equestris*, respectively, which was much lower than that from other plants, i.e., 64 for *Malus domestica* [24], 25 for *Pyrus bretschneideri* [15], 29 for *Solanum lycopersicum* [25], 32 for *A. thaliana* [4], 30 for *O. sativa* [12]. The reason for this difference might be the variable state of paralogous genes in these genomes. For example, only two *DoBBXs* paralogous and two *PeBBXs* paralogous gene pairs were identified in this study, but 18 *OsBBX* paralogous and 12 *SlBBX* paralogous gene pairs from segmental duplication events were found in *O. sativa* and *S. lycopersicum*, respectively [12, 25]. According to the phylogenetic analysis, all *BBX* genes from *A. thaliana*, *Populus trichocarpa*, *O. sativa*, *D. officinale* and *P. equestris* were clustered into five clades, which consistent with ML tree of *BBX* genes from 54 plant genomes and the previous published articles (Fig. 3b) [4]. The *BBX* gene members from *D. officinale* and *P. equestris* in clade I, II and IV had two B-BOX domains. In contrast to animal BBX with two different types of B-BOXs, the conserved amino acid sequences of the two B-BOX domains in both DoBBX and PeBBX were similar, although they are not identical. As shown in Fig. 2a, the *BBX* genes from *D. officinale*, *P. equestris* and *O. sativa* were more closely than *D. officinale* and *P. trichocarpa*. Among them, we found that the genes from clade I, II, and III, had two B-BOX domains plus a CCT domain, indicating that their might contribute to control photoperiodic regulation of flowering [2, 26]. In the clade VI and clade V subfamily, the members only had one or two B-BOX domain, but lacked CCT domain. The previous publication articles suggested that the B-BOX domain sequence in C-X_2_-C-X_8_-C-X_7_-C-X_2_-C-X_4_-H-X_8_-H in the N-terminal region, and the conservative C (Cysteine) and H (Histidine) residues were involved in BBX protein-protein [4]. In the present study, we found that the BBX members from clades I, II and IV were also contained the conservative C (Cysteine) and H (Histidine) residues. The likely evolution of *BBX* genes from *D. officinale* and *P. equestris* also occurred in other plants [20], which was supported by structure analyses of theses *BBX* genes in our study.

Gene duplications, which including whole-genome duplications, tandem duplications, transposition events and segmental duplications, contributed to genome expansion [27]. The paralogous genes from *D. officinale* and *P. equestris* cannot be distributed clearly on the chromosomes, because the chromosome assembly for *D. officinale* and *P. equestris* genomes has not yet been finished [17, 18]. Therefore, we could not authenticate the types of putative duplication events in the current study. To further understand the patterns of evolution in both *D. officinale* and *P. equestris*, the value of Ka and Ks was calculated. Particularly, we calculated the frequency distribution of the Ka and Ks for paralogous genes (Do-Do and Pe-Pe) and orthologous genes (Do-Pe), and estimated the Ks value for all homologous gene pairs. We predicted that two paralogous gene pairs (*PeBBX01*-*PeBBX07* and *DoBBX07*-*DoBBX18*) were evolved from the genome-wide duplication events by shared *D. officinale* and *P. equestris* [14], because their values of Ks were 1.0658 and 1.4562, respectively. Generally, Ka/Ks ratio greater than 1 signifies positive selection with accelerated evolution, Ka/Ks ratio equal to 1 represents neutral selection, while less than 1 means stabilizing or negative selection. Remarkably, the Ka/Ks ratios of all homologous gene pairs were less than 1, except for *DoBBX05*-*PeBBX08*, implying that these gene pairs have been experiencing a markedly purifying selection during evolution. We also noticed two homologous gene pairs (*PeBBX08*-*PeBBX14* and *DoBBX10*-*DoBBX11*) contained the comparatively high Ka/Ks values (> 0.5), suggesting that these gene pairs have undergone rapid evolutionary diversification after duplication events in the course of evolution.

The overall analysis of microarray expression profiles in different tissues will contribute to study the tissue-specific and dynamic expression of *BBX* genes in *D. officinale*. Therefore, the gene expression profiles of all 19 *BBX* genes were exhibited in *D. officinale* by using published RNA-seq data. Among them, several *BBX* genes (such as *DoBBX02*, *DoBBX03* and *DoBBX08*) presented highly expression level in eight tissues, indicating that these genes importance in the processes of *D. officinale* growth and development. Previous studies have shown that *BBX* genes play key roles in the regulation of flowering [2, 26, 28, 29], such as *A. thaliana BBX1*, *O. sativa BBX1* and *Beta vulgaris COL1*. In our study, some cis-acting elements associated to flowering were identified in *DoBBX* promoter regions, such as Skn-1-motif and GCN4_motif required for endosperm expression, and circadian for circadian control elements. The corresponding *DoBBX* genes (such as *DoBBX03* and *DoBBX12*) were also highly expressed in floral organs, indicating that these genes might important role in the formation of reproductive organs.

In the plants, many stress-associated genes could produce stress responses, which were regulated and/or mediated and by various signaling pathways [30]. The numbers of *BBX* gene family have been verified to play positive roles in abiotic stress responses and were regulated by environmental signals [12, 24]. In our study, a variety of frequently occurring cis-acting elements were identified in promoter regions of both *DoBBXs* and *PeBBXs*, such as MBS, ARE, LTR, HSF, ERE and ABRE. We also noted that these *BBX* genes contained at least one abiotic stress cis-elements, suggesting that their might contribute to responding the biotic and abiotic stresses. In order to deep understanding of stress responses mechanism in the *D. officinale BBX* genes, we performed the qRT-PCR under different treatments, such as AgNO_3_, MeJA (Methyl Jasmonate), ABA (abscisic acid) and SA (salicylic acid) in PLBs. Then, we observed the *DoBBX* genes exhibited significantly differential expression patterns under these treatments. Some *DoBBX* genes were strongly up-regulated by these treatments, indicating that these genes might play crucial roles in response abiotic stress in *D. officinale.* For example, *DoBBX17* was highly expressed (over 1700-fold that of CK levels) under ABA and *DoBBX19* was highly expressed under AgNO_3_ and MeJA treatment. Among these *DoBBX* genes, some members had a CCT domain, but the remaining members lacked CCT domains. In the current study, we found that all *DoBBX* genes responded to abiotic stress, regardless of whether they contained CCT domain. These results suggested that gene-encoded proteins having a B-BOX or CCT domain might function in response to stress. In our study, we found that *DoBBX* genes were sensitive to different abiotic stresses, such as AgNO_3_, MeJA, ABA and SA stresses. These results provided evidence that plant BBX members could participate in responding to abiotic stress responses.

## Conclusions

In our study, a comprehensive analysis of *BBX* genes was conducted in *D. officinale* and *P. equestris*, which including phylogenetic, exon-intron structure, cis-acting element, microarray analysis and qRT-PCR analysis of 19 *DoBBX* genes under various four stress treatments: AgNO_3_, MeJA (Methyl Jasmonate), ABA (abscisic acid) and SA (salicylic acid). Our experimental findings highlighted the roles in growth and development stage, and response to abiotic stress.

## Methods

### Identification of *BBX* genes in *D. officinale* and *P. equestris* genome

In our study, two different strategies were used to annotate and identify the genes encoding BBXs in *D. officinale* and *P. equestris* genome. In the first strategy, we first download the known sequences of BBXs from the TAIR database. Subsequently, we used these sequences to search the potential BBXs in *D. officinale* and *P. equestris* genome database by BLASTP program with the E value cutoff set at 1e-5. In the second strategy, we first download the HMM (hidden Markov model) profile of BBX domain (Pfam00643) from Pfam database [31]. Then this HMM profile was used to identify for all of BBXs in *D. officinale* and *P. equestris* genome by HMMER 3.0 software with the E value cutoff set at 1e-3. Finally, all putative BBX genes were further verified the presence of B-BOX domain by submitting them to InterProScan [32], Pfam [33] and SMART database [34], respectively. The ExPASY online tool was used to estimate the molecular weight and isoelectric point (pI) of all *BBX* genes [35].

### Phylogenetic analysis and sequence characterization

A total of 54 plant genomes were included in our phylogenetic analysis, including green alga (*Chlamydomonas reinhardtii*), moss (*Physcomitrella patens*), club moss (*Selaginella moellendorffii*), a single genome for gymnosperms (*Picea abies*), the early diverging angiosperm (*Amborella trichopoda*), 13 monocots, *Beta vulgaris* (non-rosid non-asterid), and 30 rosids (Additional file 1: Table S3 and Fig. 3a). Multiple sequence alignments of all BBX proteins were carried out with the MUSCLE (https://www.ebi.ac.uk/Tools/msa/muscle/) with default parameters. Subsequently, the Neighbor Joining (NJ) tree was generate by MEGA 5.2 software with bootstrap analysis (1000 replicates) [36]. The Maximum likelihood (ML) tree was generated by FastTree software with JTT model [19, 37]. The GFF3 files of individual *BBX* genes were obtained from the previously published articles, and then their gene structures were generated with GSDS website (http://gsds.cbi.pku.edu.cn/) [38]. The motif logos of the BBX and CCT domains were generated using online MEME program (http://meme.nbcr.net/meme/cgi-bin/meme.cgi) [39].

### Identification of orthologs and paralogs

Orthologs and paralogs were identified by using OrthoMCL software with the E value cutoff set at 1e-5 [40]. Based on the previous papers [41, 42], we used the DnaSP5.0 software to calculate the Ks (synonymous substitution rate), Ka (non-synonymous substitution rate) and Ka/Ks of homologous gene pairs.

### Cis-acting elements analysis of *BBX* genes in *D. officinale* and *P. equestris*

To determine the cis-acting elements, we first obtained the promoter sequences (i.e. the 1500 bp of genomic DNA sequence upstream of the initiation code (ATG)) by TBtools software. Then these promoter sequences were submitted to the PlantCARE website (http://bioinformatics.psb.ugent.be/webtools/plantcare/html/) to identify the presence of different cis-acting elements [43].

### RNA-seq expression analysis

To gain insight into the *DoBBX* gene expression patterns in different tissues of *D. officinale*, the raw RNA-seq reads from eight different tissues (root, root_tips, stem, leaf, speal, column, lip and flower_buds) were downloaded from the SRA database of NCBI (PRJNA348403). By using the HISAT2 software, the paired clean reads were mapped to the *D. officinale* reference genome with defaults parameters [44, 45]. Then the StringTie software was used to estimate the differently expressed genes [44]. The R script was used to exhibit the heatmap of *DoBBX* genes in eight different tissues (root, root_tips, stem, leaf, speal, column, lip and flower_buds).

### Plant material and stress treatments

The tissue-cultured seedlings of *D. officinale* were sterilized and planted on Murashige and Skoog (MS) (Murashige and Skoog) medium in the tissue culture room of Anhui Agricultural University under the condition of 25 °C with a constant photoperiod (16 h light/8 h dark) for about one month. Subsequently, it was transferred on MS medium supplemented with 30 g L^− 1^ sucrose (Aladdin), 0.1 mg L^− 1^ NAA (Aladdin) and 1.0 mg L^− 1^ 6-BA (biosharp). Protocorm-like bodies (PLBs) were induced from sterilized seeds and maintained on 1/2 Murashige and Skoog (MS) liquid medium supplemented with 0.1 mg/L α-naphthalene acetic acid (NAA), 0.1 g/L lactalbumin hydrolysate and 30 g/L sucrose (pH of 5.8). By liquid suspension culture with temperature 25 °C under darkness for two months, PLBs were cut into 0.5× 0.5 cm pecies, and 7 g of them were inoculated in a triangular flask containing 40 mL MS medium. In these MS medium, 100 μM MeJA (Methyl Jasmonate: Aladdin), 100 μM SA (Salicylic acid: Aladdin), 100 μM ABA (Abscisic acid: Aladdin), and 100 μM AgNO3 (Aladdin) were added after 0.22 μm microporous filtration, based on the previously published articles [46]. The PLBs were sampled at 24 h, 48 h, 72 h and 96 h after treatment. For each induction treatment, each sample (PLBs) was collected and immediately stored at − 80 °C for RNA isolation. Additionally, untreated PLBs (24 h) was used as the control group.

### Quantitative real-time PCR analysis

Total RNA from PLBs was extracted with Plant Total RNA Isolation Kit (Sangon Biotech, China) using 300 mg tissue homogenized in liquid nitrogen according to the manufacturer’s protocol, which was reverse transcribed into the first DNA strand subsequently using a One Step RT-qPCR Kit (BBI Life Science, China). The qRT-PCR was executed using 2X TaqMan Fast qPCR Master Mix (BBI Life Science, China) with CFX96 Touch™ Real-Time PCR detection system (Bio-Rad, USA) based on the manufacturer’s introduction. In the present study, each reaction contained 0.75 μl SYBR Abstart One Step RT-PCR Mix, 10 μl 2.5X SYBR One Step RT-PCR buffer, 2 μl cDNA samples, and 1 μl of each primer (10 μM) in a reaction system of 25 μl. The thermal cycle was as follows: 98 °C for 2 min, 40 cycles of 98 °C for 10 s, 60 °C for 10 s, and 68 °C for 30 s. The tubliun gene was used as an internal control [47], and the gene-specific primers (Additional file 1: Table S2) of each *DoBBX* genes were designed using Beacon Designer 7 software. Three biological replicates were carried out for each experiment.

## Additional file

Additional file 1: **Figure S1**. Sliding window analysis of Ka/Ks for the orthologous (Pe-Do) and paralogous (Pe-Pe, Do-Do) gene pairs. The window size was 150 bp, and the step size was 9 bp. **Figure S2**. Gene structures analysis of the *BBXs* in both *D. officinale* and *P. equestris*. The exons and introns are indicated by green rectangles and thin lines, respectively. **Figure S3**. Comparison of the exon-intron structure between homologous genes (Pe-Pe, Do-Do and Pe-Do). The exons and introns are indicated by green rectangles and thin lines, respectively. **Table S1**. The 19 *DoBBX* gene primer sequences. **Table S2**. The Ka, Ks and Ka/Ks values of orthologous (Pe-Do) and paralogous (Pe-Pe, Do-Do) gene pairs. **Table S3**. Plant genomes used in this analysis. (DOCX 391 kb)

## Data Availability

Expression data of *D. officinale* used in this study were available in NCBI SRA database with accession numbers of PRJNA348403.

## Abbreviations

ABA: Abscisic acid
Ka: Nonsynonymous
Ks: Synonymous
MeJA: Methyl Jasmonate
NJ: Neighbor joining
PLBs: Protocorm-like bodies
qRT-PCR: Real-Time PCR
SA: Salicylic acid
