# Supplementary material for: Comparative analysis of B-BOX genes and their expression pattern analysis under various treatments in Dendrobium officinale
Source: BMC Plant Biol. 2019 Jun 10;19:245. doi: 10.1186/s12870-019-1851-6 (PMC6558717; doi:10.1186/s12870-019-1851-6)
Supplement: Supplementary file 1 — Figure S1. Sliding window analysis of Ka/Ks for the orthologous (Pe-Do) and paralogous (Pe-Pe, Do-Do) gene pairs. The window size was 150 bp, and the step size was 9 bp. Figure S2. Gene structures analysis of the BBXs in both D. officinale and P. equestris. The exons and introns are indicated by green rectangles and thin lines, respectively. Figure S3. Comparison of the exon-intron structure between homologous genes (Pe-Pe, Do-Do and Pe-Do). The exons and introns are indicated by green rectangles and thin lines, respectively. Table S1. The 19 DoBBX gene primer sequences. Table S2. The Ka, Ks and Ka/Ks values of orthologous (Pe-Do) and paralogous (Pe-Pe, Do-Do) gene pairs. Table S3. Plant genomes used in this analysis. (DOCX 391 kb) [file 12870_2019_1851_MOESM1_ESM.docx]

**Comparative analysis of B-BOX genes and their expression pattern analysis under various treatments in *Dendrobium officinale***

**Authors:** Yunpeng Cao^#^, Yahui Han^#^, Dandan Meng^#^, Tianzhe Chen, Chunyan Jiao, Yu Chen, Muhammad Abdullah, Qing Jin, Honghong Fan, Yi Lin, Yongping Cai^*^

School of Life Sciences, Anhui Agricultural University, Hefei 230036, China.

*Corresponding author: School of Life Sciences, Anhui Agricultural University, Hefei 230036, China

Tel.: +86 551 65786137; Fax: +86 551 65786340

E-mail address: [swkx12@ahau.edu.cn](mailto:swkx12@ahau.edu.cn)

^#^These authors contributed equally to this work.


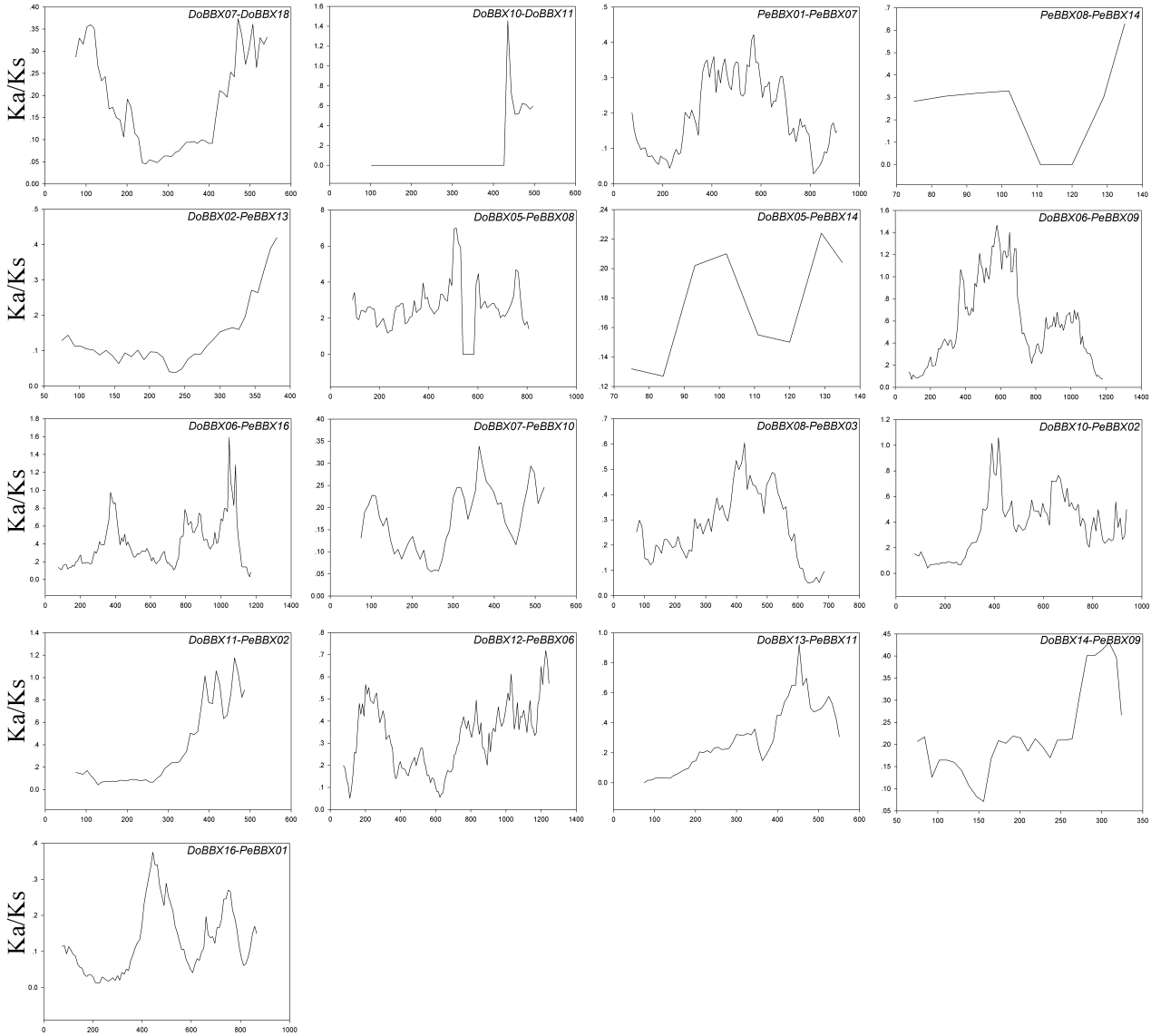


**Figure S1.** Sliding window analysis of Ka/Ks for the orthologous (Pe-Do) and paralogous (Pe-Pe, Do-Do) gene pairs. The window size was 150 bp, and the step size was 9 bp.


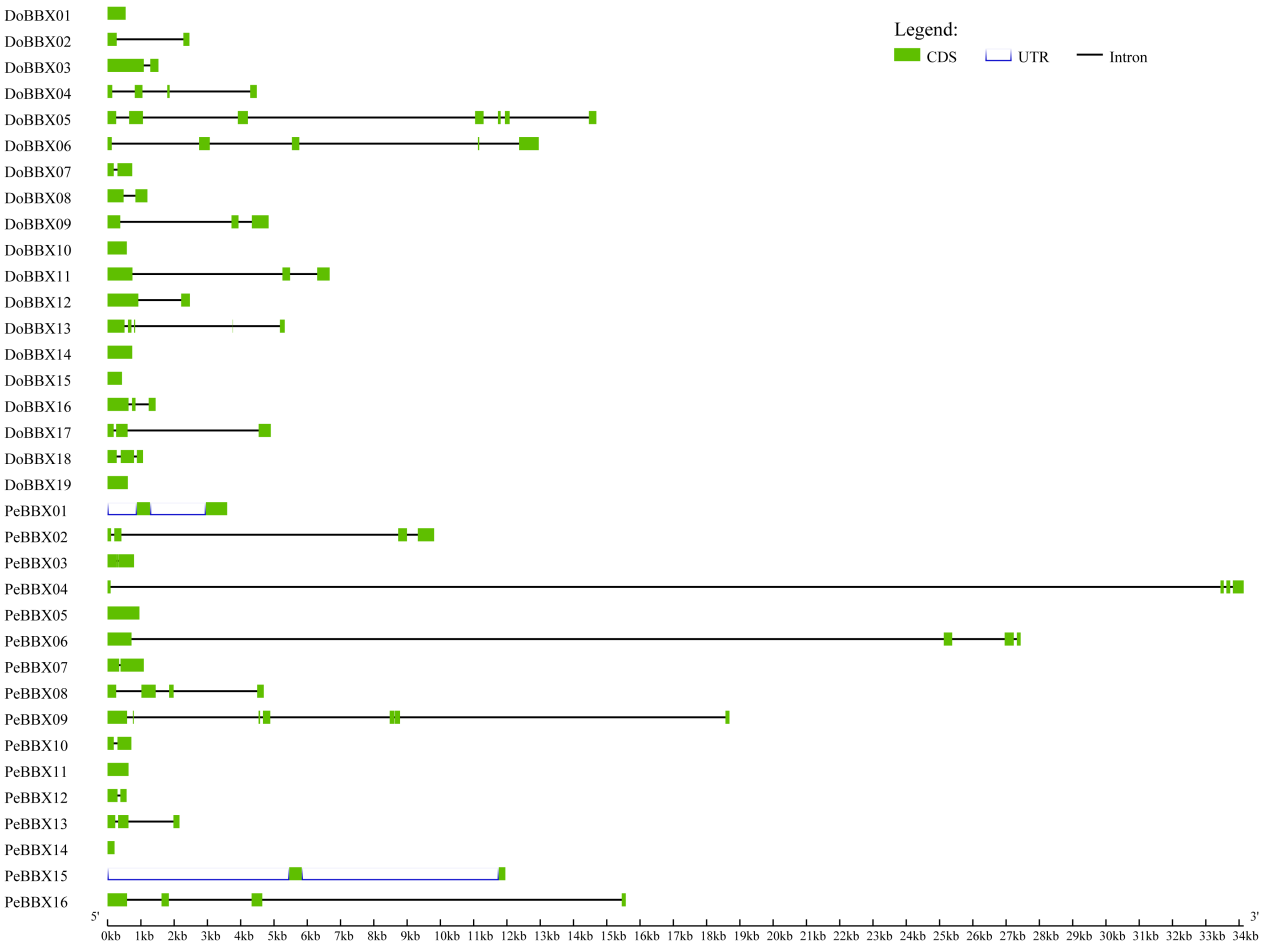


**Figure S2.** Gene structures analysis of the *BBXs* in both *D. officinale* and *P. equestris*. The exons and introns are indicated by green rectangles and thin lines, respectively.


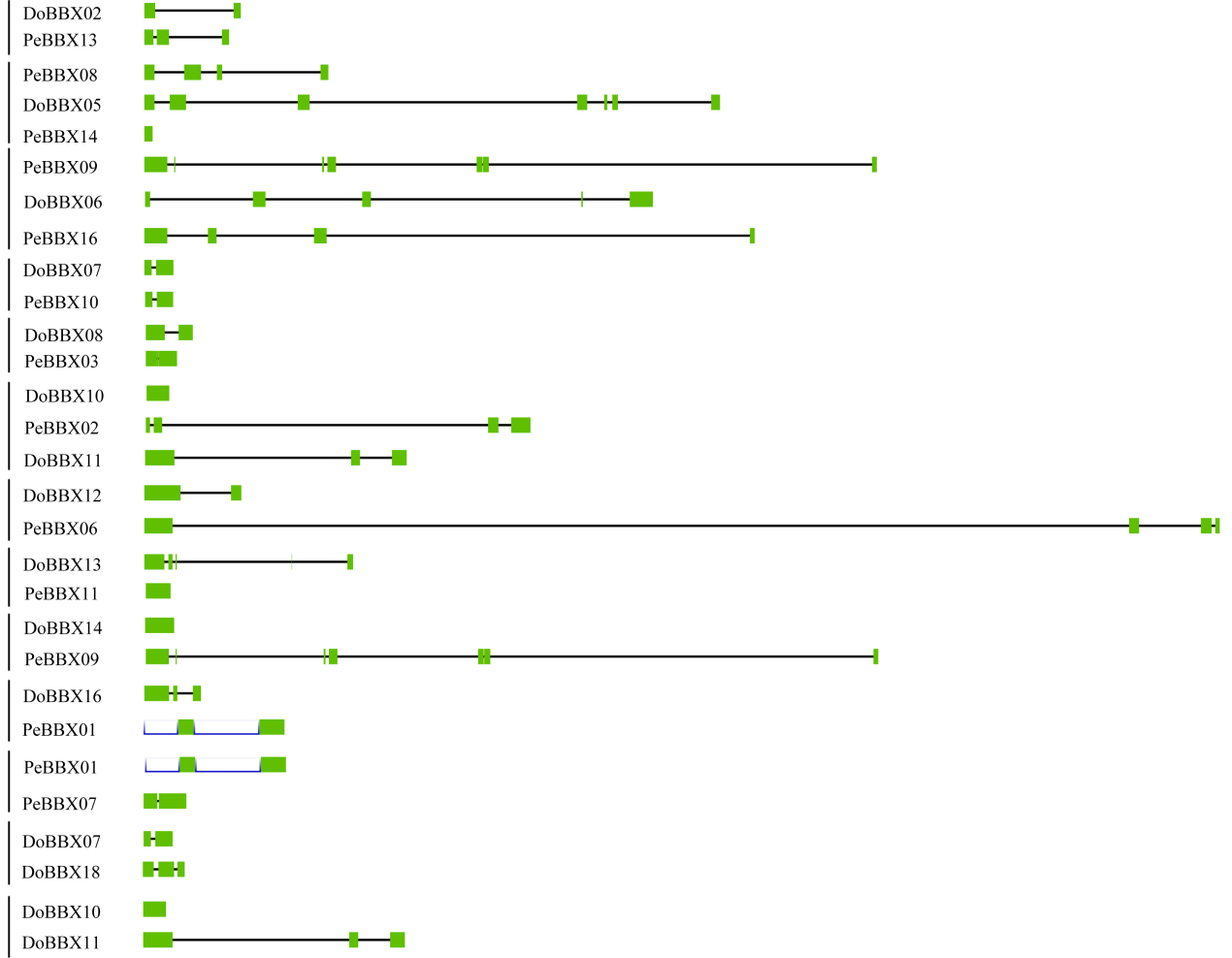


**Figure S3.** Comparison of the exon-intron structure between homologous genes (Pe-Pe, Do-Do and Pe-Do). The exons and introns are indicated by green rectangles and thin lines, respectively.

**Table S1.** The 19 *DoBBX* gene primer sequences.

| Gene pairs | Ka | Ks | Ka/Ks |
| --- | --- | --- | --- |
| *DoBBX02-PeBBX13* | 0.1058 | 0.5311 | 0.1992092 |
| *DoBBX05-PeBBX08* | 0.2224 | 0.0994 | 2.2374245 |
| *DoBBX05-PeBBX14* | 0.0775 | 0.421 | 0.1840855 |
| *DoBBX06-PeBBX09* | 0.2283 | 0.5071 | 0.4502071 |
| *DoBBX06-PeBBX16* | 0.101 | 0.3389 | 0.298023 |
| *DoBBX07-PeBBX10* | 0.0933 | 0.446 | 0.2091928 |
| *DoBBX08-PeBBX03* | 0.1847 | 0.6021 | 0.3067597 |
| *DoBBX10-PeBBX02* | 0.2889 | 0.5873 | 0.4919121 |
| *DoBBX11-PeBBX02* | 0.1296 | 0.3517 | 0.3684959 |
| *DoBBX12-PeBBX06* | 0.1518 | 0.4405 | 0.3446084 |
| *DoBBX13-PeBBX11* | 0.1731 | 0.6349 | 0.2726414 |
| *DoBBX14-PeBBX09* | 0.0629 | 0.2742 | 0.2293946 |
| *DoBBX16-PeBBX01* | 0.0796 | 0.6374 | 0.1248823 |
| *PeBBX01-PeBBX07* | 0.2263 | 1.0658 | 0.2123288 |
| *PeBBX08-PeBBX14* | 0.032 | 0.0433 | 0.73903 |
| *DoBBX07-DoBBX18* | 0.3403 | 1.4562 | 0.2336904 |
| *DoBBX10-DoBBX11* | 0.0608 | 0.0827 | 0.7351874 |

**Table S2.** The Ka, Ks and Ka/Ks values of orthologous (Pe-Do) and paralogous (Pe-Pe, Do-Do) gene pairs.

| Primer name | Sequence (5'-3') |
| --- | --- |
| DoBBX01-F | AGCATTCACTCCGCCAACA |
| DoBBX01-R | GATCATCGTGGCAGAGGAAAT |
| DoBBX02-F | TGAGGATTGGCTGCGATGT |
| DoBBX02-R | TGTTGGCTGCGTGGACTT |
| DoBBX03-F | CCGAAATCCATCCTCCATTCAC |
| DoBBX03-R | GGCTTGGGACGTTACAGGA |
| DoBBX04-F | ATGAAGATACAGTGCGATGCG |
| DoBBX04-R | GAGCGGTACTCTCTGGTGTT |
| DoBBX05-F | TGAAGGCAGGGATCATCAGG |
| DoBBX05-R | ACACCACGGCACCACATAA |
| DoBBX06-F | GTCCTTGTGCTTAGCTTGTGA |
| DoBBX06-R | ACTCGCTGGTTGTGAATAACAT |
| DoBBX07-F | GATACAGTGCGACGTGTGC |
| DoBBX07-R | GCTTGTTTGCGGAGTGGATG |
| DoBBX08-F | ACGTCATGGTGCGACTCAT |
| DoBBX08-R | GTGAGTTGGCGGAGTGGAT |
| DoBBX09-F | CGAGCGTGCGATAGCTGTAT |
| DoBBX09-R | GGGTTAGCAGAGTGGATGAGT |
| DoBBX10-F | GCCAGTTGCTTGGAGAGAA |
| DoBBX10-R | TAGAGAAAGGTGAAGTCCCTAG |
| DoBBX11-F | GCAGGTGTTTAGCTGGGATG |
| DoBBX11-R | AGGCGGAGGTTCCTTCGTA |
| DoBBX12-F | ATGAGGAGAGTACAGGTGAGC |
| DoBBX12-R | ACCGCATTTGTCGCAGATC |
| DoBBX13-F | CAGCGAGGATGTATTGCGAATC |
| DoBBX13-R | AAGGCGTCTGAGCTTGGCA |
| DoBBX14-F | GCATCCCTTTGCTTGTCTTG |
| DoBBX14-R | CTAATTGTCGCAGGCTGTGA |
| DoBBX15-F | AATCAGACCAGGCGATACTGT |
| DoBBX15-R | TGGCATCTCCGGCATAGAAG |
| DoBBX16-F | GAGGGAAAGGAGCAGAGGGT |
| DoBBX16-R | AGTTTGTTCGCGCCGTGCA |
| DoBBX17-F | ATGAAGCTACTCTGCGATGTT |
| DoBBX17-R | GAGGGTGTGTTTGAGTGTAGG |
| DoBBX18-F | ATGAAGATACAGTGCGACGT |
| DoBBX18-R | CGAGCTTATTGGCGGAGTG |
| DoBBX19-F | GAATCAGACCAGGCGATACT |
| DoBBX19-R | TTTCATAGCTCCCTCAATATGG |

**Table S3. Plant genomes used in this analysis**

| **No** | **Species** | **Order** | **Version** | **Genes** | **Reference** |
| --- | --- | --- | --- | --- | --- |
| 1 | *Phaseolus vulgaris*(Common bean) | Rosids | Version 1.0 | 27082 | Schmutz et al., 2014 |
| 2 | *Glycine max*(Soybean) | Rosids | Wm82.a2.v1 | 56044 | Schmutz et al., 2010 |
| 3 | *Cajanus cajan*(Pigeonpea) | Rosids | Nov_2011 | 48680 | Varshney et al., 2012 |
| 4 | *Medicago truncatula*(Barrel medic) | Rosids | Mt4.0v1 | 50894 | Young et al., 2011 |
| 5 | *Cicer arietinum*(Chickpea) | Rosids | Version 1.0 | 28269 | Varshney et al., 2013 |
| 6 | *Lotus japonicus*(Lotus) | Rosids | Version 2.5 | 42399 | Sato et al., 2008 |
| 7 | *Citrullus lanatus*(Watermelon) | Rosids | Version 1.0 | 23440 | Guo et al., 2013 |
| 8 | *Cucumis sativus*(Cucumber) | Rosids | Version 1.0 | 21491 | Huang et al., 2009 |
| 9 | *Populus trichocarpa*(Western poplar) | Rosids | Version 3.0 | 41335 | Tuskan et al., 2006 |
| 10 | *Ricinus communis* (Castor bean) | Rosids | Version 0.1 | 38613 | Chan et al., 2010 |
| 11 | *Malus x domestica*(Apple) | Rosids | Version 1.0 | 63514 | Velasco et al., 2010 |
| 12 | *Pyrus x bretschneideri* (Pear) | Rosids | Version 1.0 | 42812 | Wu et al., 2013 |
| 13 | *Prunus persica*(Peach) | Rosids | Version 1.0 | 28689 | International Peach Genome et al., 2013 |
| 14 | *Prunus mume* (Mei) | Rosids | Version 1.0 | 31390 | Zhang et al., 2012 |
| 15 | *Fragaria vesca* (Strawberry) | Rosids | Version 1.1 | 32831 | Shulaev et al., 2011 |
| 16 | *Arabidopsis thaliana*(Arabidopsis) | Rosids | TAIR10 | 27416 | Arabidopsis Genome, 2000 |
| 17 | *Arabidopsis lyrata*(Lyrate rockcress) | Rosids | Version 1.0 | 32670 | Hu et al., 2011 |
| 18 | *Capsella rubella*(Capsella) | Rosids | Version 1.0 | 26521 | Slotte et al., 2013 |
| 19 | *Brassica oleracea* (Kale) | Rosids | Version 2.1 | 59225 | Liu et al., 2014 |
| 20 | *Brassica rapa*(Chinese cabbage) | Rosids | Version 1.3 | 40492 | Wang et al., 2011 |
| 21 | *Aethionema* | Rosids | Version 2.5 | 22230 | Haudry et al., 2013 |
| 22 | *Tarenaya* | Rosids | Version 5 | 31580 | Cheng et al., 2013 |
| 23 | *Carica papaya*(Papaya) | Rosids | ASGPBv0.4 | 24782 | Ming et al., 2008 |
| 24 | *Gossypium raimondii*(Cotton) | Rosids | Version 2.1 | 37505 | Paterson et al., 2012 |
| 25 | *Theobroma cacao* (Cacao) | Rosids | Version 1.1 | 29452 | Argout et al., 2011 |
| 26 | *Citrus sinensis* (Sweet orange) | Rosids | Version 1.1 | 25379 | Xu et al., 2013 |
| 27 | *Eucalyptus grandis*(Eucalyptus) | Rosids | Version 1.1 | 36376 | Myburg et al., 2014 |
| 28 | *Vitis vinifera*(Grape vine) | Rosids | Genoscope (Aug 2007) | 26346 | Jaillon et al., 2007 |
| 29 | *Solanum tuberosum*(Potato) | Solanace | Version 3.4 | 39031 | Potato Genome Sequencing et al., 2011 |
| 30 | *Solanum lycopersicum*(Tomato) | Solanace | Version 2.4 | 34727 | Tomato Genome, 2012 |
| 31 | *Capsicum annuum* (Hot pepper) | Solanace | Version 1.55 | 34899 | Kim et al., 2014 |
| 32 | *Utricularia gibba*(Humped bladderwort) | Solanace | CoGe (Jun 2013) | 28494 | Ibarra-Laclette et al., 2013 |
| 33 | *Actinidia chinensis*(Kiwifruit) | Solanace | May_2013 | 32670 | Huang et al., 2013 |
| 34 | *Beta vulgaris*(Sugar beet) | Eudicots | RefBeet-1.1 | 27421 | Dohm et al., 2014 |
| 35 | *Nelumbo nucifera* (Sacred lotus) | Eudicots | Version 1.0 | 26685 | Ming et al., 2013 |
| 36 | *Triticum urartu*(Wheat A-genome) | Monocots | Version 1.0 | 34879 | Ling et al., 2013 |
| 37 | *Hordeum vulgare* (Barley) | Monocots | Version 1.0 | 16598 | International Barley Genome Sequencing et al., 2012 |
| 38 | *Brachypodium distachyon*(Purple false brome) | Monocots | Version 2.1 | 31694 | International Brachypodium, 2010 |
| 39 | *Oryza sativa*(Rice) | Monocots | Version 7.0 | 39049 | International Rice Genome Sequencing, 2005 |
| 40 | *Zea mays*(Maize) | Monocots | Version 6a | 63480 | Schnable et al., 2009 |
| 41 | *Sorghum bicolor*(Sorghum) | Monocots | Version 2.1 | 33032 | Paterson et al., 2009 |
| 42 | *Setaria italica* | Monocots | Version 2.1 | 35471 | Bennetzen et al., 2012 |
| 43 | *Elaeis guineensis* (Oil palm) | Monocots | Version 2.0 | 30752 | Singh et al., 2013 |
| 44 | *Musa acuminata* (Banana) | Monocots | July_2012 | 36542 | D'Hont et al., 2012 |
| 45 | *Phalaenopsis equestris*(Orchid) | Monocots | Version 5.0 | 42293 | Cai et al., 2015 |
| 46 | *Apostasia shenzhenica*(Orchid) | Monocots | Version 1.0 | 21841 | Zhang et al., 2017 |
| 47 | *Gastrodia elata*(Orchid) | Monocots | Version 1.0 | 18969 | Yuan et al., 2018 |
| 48 | *Dendrobium officinale*(Orchid) | Monocots | Version 1.0 | 28910 | Yan et al., 2015 |
| 49 | *Zostera muelleri*(Seagrass) | Monocots | Version 1.0 | 33245 | Golicz et al., 2015 |
| 50 | *Amborella trichopoda*(Amborella) | Basal Angiosperm | Version 1.0 | 26846 | Chamala et al., 2013 |
| 51 | *Picea abies*(Norway spruce) | Gymnosperm | Version 1.0 | 66632 | Nystedt et al., 2013 |
| 52 | *Selaginella moellendorffii* (Selaginella) | Moss | Version 1.0 | 22273 | Banks et al., 2011 |
| 53 | *Physcomitrella patens* (Moss) | Moss | Version 3.0 | 26610 | Rensing et al., 2008 |
| 54 | *Chlamydomonas reinhardtii*(Green algae) | Green algae | Version 5.5 | 17741 | Merchant et al., 2007 |

**References**

Arabidopsis Genome, I. (2000). Analysis of the genome sequence of the flowering plant Arabidopsis thaliana. Nature 408, 796-815.

Argout, X., Salse, J., Aury, J.M., Guiltinan, M.J., Droc, G., Gouzy, J., Allegre, M., Chaparro, C., Legavre, T., Maximova, S.N., et al. (2011). The genome of Theobroma cacao. Nat Genet 43, 101-108.

Banks, J.A., Nishiyama, T., Hasebe, M., Bowman, J.L., Gribskov, M., dePamphilis, C., Albert, V.A., Aono, N., Aoyama, T., Ambrose, B.A., et al. (2011). The Selaginella genome identifies genetic changes associated with the evolution of vascular plants. Science 332, 960-963.

Bennetzen, J.L., Schmutz, J., Wang, H., Percifield, R., Hawkins, J., Pontaroli, A.C., Estep, M., Feng, L., Vaughn, J.N., Grimwood, J., et al. (2012). Reference genome sequence of the model plant Setaria. Nature biotechnology 30, 555-561.

Cai, J., Liu, X., Vanneste, K., Proost, S., Tsai, W.C., Liu, K.W., Chen, L.J., He, Y., Xu, Q., Bian, C., et al. (2015). The genome sequence of the orchid Phalaenopsis equestris. Nat Genet 47, 65-72.

Chamala, S., Chanderbali, A.S., Der, J.P., Lan, T.Y., Walts, B., Albert, V.A., Depamphilis, C.W., Leebens-Mack, J., Rounsley, S., Schuster, S.C., et al. (2013). Assembly and validation of the genome of the nonmodel basal angiosperm Amborella. Science 342, 1516-1517.

Chan, A.P., Crabtree, J., Zhao, Q., Lorenzi, H., Orvis, J., Puiu, D., Melake-Berhan, A., Jones, K.M., Redman, J., Chen, G., et al. (2010). Draft genome sequence of the oilseed species Ricinus communis. Nature biotechnology 28, 951-956.

Cheng, S.F., van den Bergh, E., Zeng, P., Zhong, X., Xu, J.J., Liu, X., Hofberger, J., de Bruijn, S., Bhide, A.S., Kuelahoglu, C., et al. (2013). The Tarenaya hassleriana genome provides insight into reproductive trait and genome evolution of crucifers. The Plant cell 25, 2813-2830.

D'Hont, A., Denoeud, F., Aury, J.M., Baurens, F.C., Carreel, F., Garsmeur, O., Noel, B., Bocs, S., Droc, G., Rouard, M., et al. (2012). The banana (Musa acuminata) genome and the evolution of monocotyledonous plants. Nature 488, 213-217.

Dohm, J.C., Minoche, A.E., Holtgrawe, D., Capella-Gutierrez, S., Zakrzewski, F., Tafer, H., Rupp, O., Sorensen, T., Stracke, R., Reinhardt, R., et al. (2014). The genome of the recently domesticated crop plant sugar beet (Beta vulgaris). Nature 505, 546-+.

Golicz, A.A., Schliep, M., Lee, H.T., Larkum, A.W.D., Dolferus, R., Batley, J., Chan, C.K.K., Sablok, G., Ralph, P.J., and Edwards, D. (2015). Genome-wide survey of the seagrass Zostera muelleri suggests modification of the ethylene signalling network. J Exp Bot 66, 1489-1498.

Guo, S., Zhang, J., Sun, H., Salse, J., Lucas, W.J., Zhang, H., Zheng, Y., Mao, L., Ren, Y., Wang, Z., et al. (2013). The draft genome of watermelon (Citrullus lanatus) and resequencing of 20 diverse accessions. Nat Genet 45, 51-58.

Haudry, A., Platts, A.E., Vello, E., Hoen, D.R., Leclercq, M., Williamson, R.J., Forczek, E., Joly-Lopez, Z., Steffen, J.G., Hazzouri, K.M., et al. (2013).

An atlas of over 90,000 conserved noncoding sequences provides insight into crucifer regulatory regions. Nat Genet 45, 891-U228.

Hu, T.T., Pattyn, P., Bakker, E.G., Cao, J., Cheng, J.F., Clark, R.M., Fahlgren, N., Fawcett, J.A., Grimwood, J., Gundlach, H., et al. (2011). The Arabidopsis lyrata genome sequence and the basis of rapid genome size change. Nat Genet 43, 476-481.

Huang, S., Ding, J., Deng, D., Tang, W., Sun, H., Liu, D., Zhang, L., Niu, X., Zhang, X., Meng, M., et al. (2013). Draft genome of the kiwifruit Actinidia chinensis. Nature communications 4, 2640.

Huang, S., Li, R., Zhang, Z., Li, L., Gu, X., Fan, W., Lucas, W.J., Wang, X., Xie, B., Ni, P., et al. (2009). The genome of the cucumber, Cucumis sativus L. Nat Genet 41, 1275-1281.

Ibarra-Laclette, E., Lyons, E., Hernandez-Guzman, G., Perez-Torres, C.A., Carretero-Paulet, L., Chang, T.H., Lan, T., Welch, A.J., Juarez, M.J., Simpson, J., et al. (2013). Architecture and evolution of a minute plant genome. Nature 498, 94-98.

International Barley Genome Sequencing, C., Mayer, K.F., Waugh, R., Brown, J.W., Schulman, A., Langridge, P., Platzer, M., Fincher, G.B., Muehlbauer, G.J., Sato, K., et al. (2012). A physical, genetic and functional sequence assembly of the barley genome. Nature 491, 711-716.

International Brachypodium, I. (2010). Genome sequencing and analysis of the model grass Brachypodium distachyon. Nature 463, 763-768.

International Peach Genome, I., Verde, I., Abbott, A.G., Scalabrin, S., Jung, S., Shu, S., Marroni, F., Zhebentyayeva, T., Dettori, M.T., Grimwood, J., et al. (2013). The high-quality draft genome of peach (Prunus persica) identifies unique patterns of genetic diversity, domestication and genome evolution. Nat Genet 45, 487-494.

International Rice Genome Sequencing, P. (2005). The map-based sequence of the rice genome. Nature 436, 793-800.

Jaillon, O., Aury, J.M., Noel, B., Policriti, A., Clepet, C., Casagrande, A., Choisne, N., Aubourg, S., Vitulo, N., Jubin, C., et al. (2007). The grapevine genome sequence suggests ancestral hexaploidization in major angiosperm phyla. Nature 449, 463-467.

Kim, S., Park, M., Yeom, S.I., Kim, Y.M., Lee, J.M., Lee, H.A., Seo, E., Choi, J., Cheong, K., Kim, K.T., et al. (2014). Genome sequence of the hot pepper provides insights into the evolution of pungency in Capsicum species. Nat Genet 46, 270-278.

Ling, H.Q., Zhao, S., Liu, D., Wang, J., Sun, H., Zhang, C., Fan, H., Li, D., Dong, L., Tao, Y., et al. (2013). Draft genome of the wheat A-genome progenitor Triticum urartu. Nature 496, 87-90.

Liu, S., Liu, Y., Yang, X., Tong, C., Edwards, D., Parkin, I.A., Zhao, M., Ma, J., Yu, J., Huang, S., et al. (2014). The Brassica oleracea genome reveals the asymmetrical evolution of polyploid genomes. Nature communications 5, 3930.

Merchant, S.S., Prochnik, S.E., Vallon, O., Harris, E.H., Karpowicz, S.J., Witman, G.B., Terry, A., Salamov, A., Fritz-Laylin, L.K., Marechal-Drouard, L.,et al. (2007). The Chlamydomonas genome reveals the evolution of key animal and plant functions. Science 318, 245-250.

Ming, R., Hou, S., Feng, Y., Yu, Q., Dionne-Laporte, A., Saw, J.H., Senin, P., Wang, W., Ly, B.V., Lewis, K.L., et al. (2008). The draft genome of thetransgenic tropical fruit tree papaya (Carica papaya Linnaeus). Nature 452, 991-996.

Ming, R., VanBuren, R., Liu, Y.L., Yang, M., Han, Y.P., Li, L.T., Zhang, Q., Kim, M.J., Schatz, M.C., Campbell, M., et al. (2013). Genome of the long-living sacred lotus (Nelumbo nucifera Gaertn.). Genome biology 14.

Myburg, A.A., Grattapaglia, D., Tuskan, G.A., Hellsten, U., Hayes, R.D., Grimwood, J., Jenkins, J., Lindquist, E., Tice, H., and Bauer, D. (2014). The genome of Eucalyptus grandis. Nature 510, 356-362.

Nystedt, B., Street, N.R., Wetterbom, A., Zuccolo, A., Lin, Y.C., Scofield, D.G., Vezzi, F., Delhomme, N., Giacomello, S., Alexeyenko, A., et al. (2013). The Norway spruce genome sequence and conifer genome evolution. Nature 497, 579-584.

Paterson, A.H., Bowers, J.E., Bruggmann, R., Dubchak, I., Grimwood, J., Gundlach, H., Haberer, G., Hellsten, U., Mitros, T., Poliakov, A., et al. (2009). The Sorghum bicolor genome and the diversification of grasses. Nature 457, 551-556.

Paterson, A.H., Wendel, J.F., Gundlach, H., Guo, H., Jenkins, J., Jin, D., Llewellyn, D., Showmaker, K.C., Shu, S., Udall, J., et al. (2012). Repeated polyploidization of Gossypium genomes and the evolution of spinnable cotton fibres. Nature 492, 423-427.

Potato Genome Sequencing, C., Xu, X., Pan, S., Cheng, S., Zhang, B., Mu, D., Ni, P., Zhang, G., Yang, S., Li, R., et al. (2011). Genome sequence and analysis of the tuber crop potato. Nature 475, 189-195.

Rensing, S.A., Lang, D., Zimmer, A.D., Terry, A., Salamov, A., Shapiro, H., Nishiyama, T., Perroud, P.F., Lindquist, E.A., Kamisugi, Y., et al. (2008). The Physcomitrella genome reveals evolutionary insights into the conquest of land by plants. Science 319, 64-69.

Sato, S., Nakamura, Y., Kaneko, T., Asamizu, E., Kato, T., Nakao, M., Sasamoto, S., Watanabe, A., Ono, A., Kawashima, K., et al. (2008). Genome structure of the legume, Lotus japonicus. DNA research 15, 227-239.

Schmutz, J., Cannon, S.B., Schlueter, J., Ma, J., Mitros, T., Nelson, W., Hyten, D.L., Song, Q., Thelen, J.J., Cheng, J., et al. (2010). Genome sequence of the palaeopolyploid soybean. Nature 463, 178-183.

Schmutz, J., McClean, P.E., Mamidi, S., Wu, G.A., Cannon, S.B., Grimwood, J., Jenkins, J., Shu, S., Song, Q., Chavarro, C., et al. (2014). A reference genome for common bean and genome-wide analysis of dual domestications. Nat Genet 46, 707-713.

Schnable, P.S., Ware, D., Fulton, R.S., Stein, J.C., Wei, F., Pasternak, S., Liang, C., Zhang, J., Fulton, L., Graves, T.A., et al. (2009). The B73 maize genome: complexity, diversity, and dynamics. Science 326, 1112-1115.

Shulaev, V., Sargent, D.J., Crowhurst, R.N., Mockler, T.C., Folkerts, O., Delcher, A.L., Jaiswal, P., Mockaitis, K., Liston, A., Mane, S.P., et al. (2011). The genome of woodland strawberry (Fragaria vesca). Nat Genet 43, 109-116.

Singh, R., Ong-Abdullah, M., Low, E.T., Manaf, M.A., Rosli, R., Nookiah, R., Ooi, L.C., Ooi, S.E., Chan, K.L., Halim, M.A., et al. (2013). Oil palm genome sequence reveals divergence of interfertile species in Old and New worlds. Nature 500, 335-339.

Slotte, T., Hazzouri, K.M., Agren, J.A., Koenig, D., Maumus, F., Guo, Y.L., Steige, K., Platts, A.E., Escobar, J.S., Newman, L.K., et al. (2013). The Capsella rubella genome and the genomic consequences of rapid mating system evolution. Nat Genet 45, 831-835.

Tomato Genome, C. (2012). The tomato genome sequence provides insights into fleshy fruit evolution. Nature 485, 635-641.

Tuskan, G.A., Difazio, S., Jansson, S., Bohlmann, J., Grigoriev, I., Hellsten, U., Putnam, N., Ralph, S., Rombauts, S., Salamov, A., et al. (2006). The genome of black cottonwood, Populus trichocarpa (Torr. & Gray). Science 313, 1596-1604.

Varshney, R.K., Chen, W., Li, Y., Bharti, A.K., Saxena, R.K., Schlueter, J.A., Donoghue, M.T., Azam, S., Fan, G., Whaley, A.M., et al. (2012). Draft genome sequence of pigeonpea (Cajanus cajan), an orphan legume crop of resource-poor farmers. Nature biotechnology 30, 83-89.

Varshney, R.K., Song, C., Saxena, R.K., Azam, S., Yu, S., Sharpe, A.G., Cannon, S., Baek, J., Rosen, B.D., Tar'an, B., et al. (2013). Draft genome sequence of chickpea (Cicer arietinum) provides a resource for trait improvement. Nature biotechnology 31, 240-246.

Velasco, R., Zharkikh, A., Affourtit, J., Dhingra, A., Cestaro, A., Kalyanaraman, A., Fontana, P., Bhatnagar, S.K., Troggio, M., Pruss, D., et al. (2010). The genome of the domesticated apple (Malus x domestica Borkh.). Nat Genet 42, 833-839.

Wang, X., Wang, H., Wang, J., Sun, R., Wu, J., Liu, S., Bai, Y., Mun, J.H., Bancroft, I., Cheng, F., et al. (2011). The genome of the mesopolyploid crop species Brassica rapa. Nat Genet 43, 1035-1039.

Wu, J., Wang, Z., Shi, Z., Zhang, S., Ming, R., Zhu, S., Khan, M.A., Tao, S., Korban, S.S., Wang, H., et al. (2013). The genome of the pear (Pyrus bretschneideri Rehd.). Genome research 23, 396-408.

Xu, Q., Chen, L.-L., Ruan, X., Chen, D., Zhu, A., Chen, C., Bertrand, D., Jiao, W.-B., Hao, B.-H., and Lyon, M.P. (2013). The draft genome of sweet orange (Citrus sinensis). Nat Genet 45, 59-66.

Young, N.D., Debelle, F., Oldroyd, G.E., Geurts, R., Cannon, S.B., Udvardi, M.K., Benedito, V.A., Mayer, K.F., Gouzy, J., Schoof, H., et al. (2011). The Medicago genome provides insight into the evolution of rhizobial symbioses. Nature 480, 520-524.

Zhang, Q., Chen, W., Sun, L., Zhao, F., Huang, B., Yang, W., Tao, Y., Wang, J., Yuan, Z., Fan, G., et al. (2012). The genome of Prunus mume. Nature communications 3, 1318.
